# Merge and separation of NuA4 and SWR1 complexes control cell fate plasticity in *Candida albicans*

**DOI:** 10.1038/s41421-018-0043-0

**Published:** 2018-08-14

**Authors:** Xiongjun Wang, Wencheng Zhu, Peng Chang, Hongyu Wu, Haoping Liu, Jiangye Chen

**Affiliations:** 1 State Key Laboratory of Molecular Biology, Institute of Biochemistry and Cell Biology, Shanghai Institutes for Biological Sciences, Chinese Academy of Sciences, 320 Yue Yang Road, Shanghai, 200031 China; 2 Department of Biological Chemistry, University of California, Irvine, CA 92697 USA

## Abstract

Phenotypic plasticity is common in development. *Candida albicans*, a polymorphic fungal pathogen of humans, possesses the unique ability to achieve rapid and reversible cell fate between unicellular form (yeast) and multicellular form (hypha) in response to environmental cues. The NuA4 histone acetyltransferase activity and Hda1 histone deacetylase activity have been reported to be required for hyphal initiation and maintenance. However, how Hda1 and NuA4 regulate hyphal elongation is not clear. NuA4 histone acetyltransferase and SWR1 chromatin remodeling complexes are conserved from yeast to human, which may have merged together to form a larger TIP60 complex since the origin of metazoan. In this study, we show a dynamic merge and separation of NuA4 and SWR1 complexes in *C. albicans*. NuA4 and SWR1 merge together in yeast state and separate into two distinct complexes in hyphal state. We demonstrate that acetylation of Eaf1 K173 controls the interaction between the two complexes. The YEATS domain of Yaf9 in *C. albicans* can recognize an acetyl-lysine of the Eaf1 and mediate the Yaf9-Eaf1 interaction. The reversible acetylation and deacetylation of Eaf1 by Esa1 and Hda1 control the merge and separation of NuA4 and SWR1, and this regulation is triggered by Brg1 recruitment of Hda1 to chromatin in response nutritional signals that sustain hyphal elongation. We have also observed an orchestrated promoter association of Esa1, Hda1, Swr1, and H2A.Z during the reversible yeast–hyphae transitions. This is the first discovery of a regulated merge of the NuA4 and SWR1 complexes that controls cell fate determination and this regulation may be conserved in polymorphic fungi.

## Introduction

Cell fate plasticity is common in a broad range of biological events from embryo development to tissue regeneration. Reprogramming of cell fate is regulated in multiple layers including epigenetic modifications at both DNA and chromatin levels^1,2^. Chromatin structure can be altered through a variety of mechanisms in response to extracellular signals, which results in the change of gene expression and reflects cell type specification. Posttranslational modifications of histones and chromatin remodeling are two major ways to modulate chromatin structure and have important roles in cell fate determination and conversion.

**Fig. 1:**
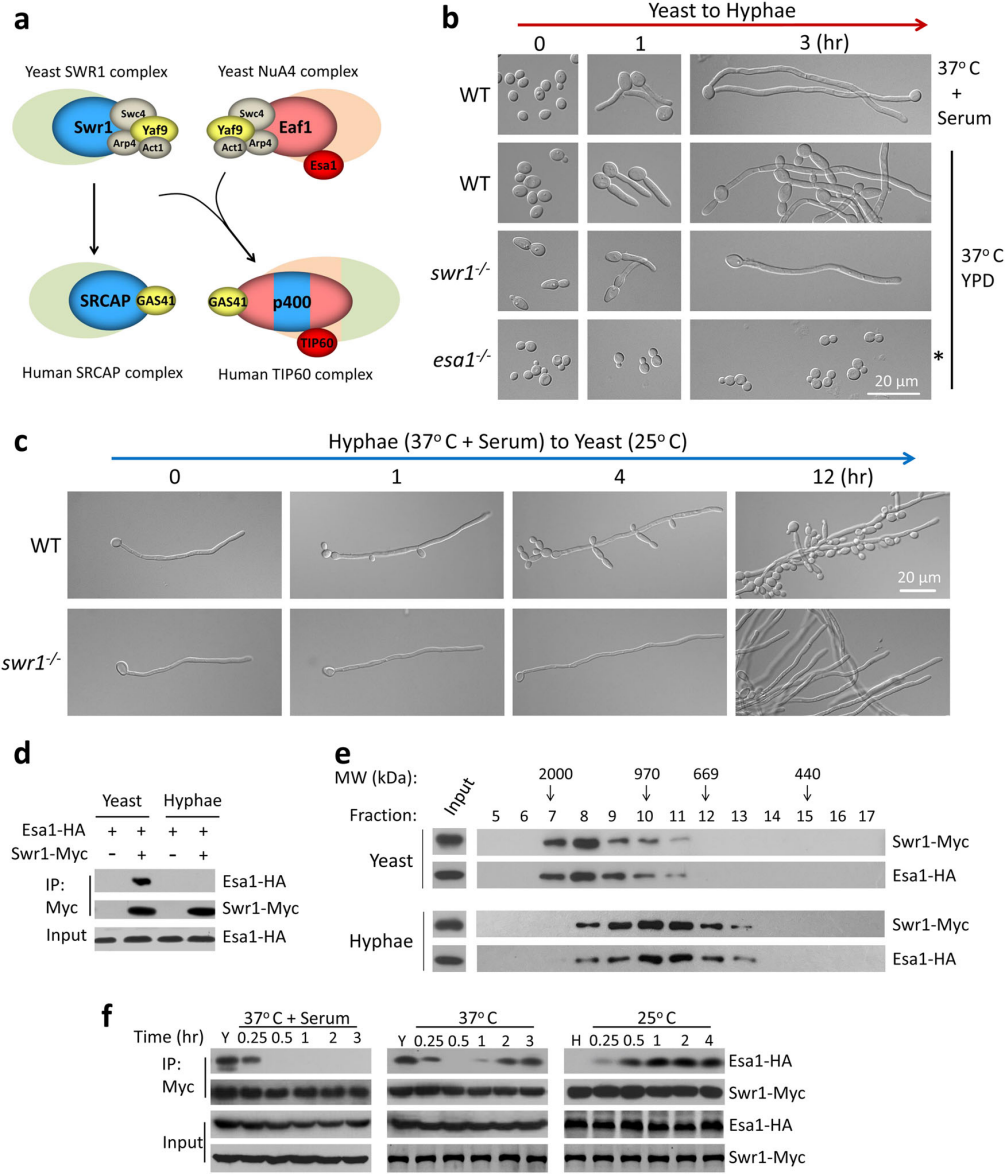
Merge and separation of NuA4 and SWR1 complexes are correlated with the reversible yeast–hypha transition in *Candida albicans*. **a** Schematic diagram showing a proposed model for the evolution of NuA4 and SWR1 complexes from yeast to human. **b** Swr1 and Esa1 have opposite roles during hyphal development. An overnight culture of wild-type (SC5314) yeast cells were diluted 1:100 into fresh YPD medium and induced at 37 °C in the presence or absence of 10% serum. The *swr1* mutant was induced at 37 °C and *esa1* mutant at 35 °C (asterisked) in YPD without serum. Cell morphoplogy was observed at 0 h, 1 h and 3 h. **d** Hyphae-to-yeast transition. The induced hyphae of wild-type and *swr1* mutant (in YPD with 10% serum at 37 °C for 3 h) were re-cultured in fresh YPD at 25 °C for indicated time. **d**, **e** Esa1 and Swr1 associated in yeast and dissociated in hyphae. A wild-type strain (BWP17) carrying Esa1-HA and Swr1-Myc under their endogenous promoters was cultured in YPD at 25 °C for 12 h to OD_600_ ~3 (yeast state) or YPD with 10% serum at 37 °C for 3 h (hyphae state). Whole-cell extracts (WCEs) of yeast or hyphae were subjected for co-immunoprecipation (Co-IP) (**d**) or Gel filtration (**e**) experiments. WCEs were immunoprecipitated with anti-Myc antibody and probed with anti-HA or anti-Myc (**d**). Every second fraction eluted from a Superose 6 column was analyzed for the presence of Swr1-Myc, together with Esa1-HA by western blotting (**e**). Native molecular weight markers eluting in the corresponding fractions are indicated on the top of the panel. **f** Esa1-Swr1 association during reversible yeast–hypha transition. The cells were cultured in conditions described and collected at time points indicated for Co-IP. Western blot analysis was carried out using a peroxidase-conjugated anti-Myc antibody or anti-HA antibody to assess levels of Swr1-Myc or Esa1-HA

NuA4 (nucleosome acetyltransferase of H4) is a multi-subunit HAT (histone acetyltransferase) complex that is highly conserved in eukaryotes and has important roles in cell cycle progression, cell transformation, development, apoptosis, transcription, and DNA repair^3–9^. *Saccharomyces cerevisiae* NuA4 is composed of 13 subunits^9,10^, including the essential acetyltransferase subunit Esa1^11–13^ and the platform protein Eaf1, which has a crucial function in NuA4 complex integrity and assembly^14,15^ (Fig.1a). *S*. *cerevisiae* SWR1 is the first complex identified as an ATP-dependent remodeling complex for deposition of histone variant H2A.Z^16-18^. SWR1 is composed of 14 subunits organized into discrete functional modules. The core subunit Swr1 is a platform protein with several distinct domains, such as the HSA (helicase/SANT-associated) and SWI2/SNF2 family ATPase domains that are involved in diverse aspects of SWR1 function. Beyond its catalytic activity, Swr1 also acts as a scaffold for assembly of numerous SWR1 components and mediates a limited interaction with the nucleosome^5,19^. A four-component module, Yaf9-Arp4-Swc4-Act1 is shared by both SWR1 and NuA4 complexes in *S. cerevisiae* and functions at the center of their regulatory circuitry^5,20^. The HSA domain contained in the two platform proteins, Swr1 and Eaf1, mediates the interaction within the shared four-component module^10,14,15,19^ (Fig. 1a).

In human, homologs of yeast NuA4 and SWR1 subunits form a hybrid complex called TIP60, which can acetylate histones H2A and H4, and exchange H2A with H2A.Z in vitro^9,21–23^. p400, a platform crucial for integrity of the TIP60 complex, combines the features of Eaf1 and Swr1 and contains an HSA, ATPase, and SANT domain. In addition to the TIP60 complex, SRCAP functions as the human counterpart of the yeast SWR1 to deposit H2A.Z into chromatin^24,25^. The TIP60 and SRCAP complexes share several subunits, such as the counterpart of the yeast four-component module (Fig. 1a). Although the two complexes have an overlapping basic function to deposit H2A.Z into chromatin, they appear to regulate distinct processes^5,23,26^. Interestingly, p400-like proteins are found broadly in metazoan and plant (http://blast.ncbi.nlm.nih.gov/Blast.cgi), but not in fungi. The significance of the merge between the NuA4 and SWR1 complexes in higher eukaryotes from two distinct complexes in the fungal kingdom in evolution is not clear.

*Candida albicans* is a polymorphic fungal pathogen of humans, possessing the unique ability to switch reversibly between unicellular budding yeast and multicellular filamentous form (hypha) in response to environmental cues^27,28^. Hyphal development requires two sequential regulations of the promoter chromatin of hypha-specific genes^28^. Hyphal initiation requires a rapid but temporary disappearance of the hyphal transcriptional repressor Nrg1, leading to dissociation of the Rpd3 histone deacetylase from the promoters, an increase in promoter chromatin H4 acetylation by NuA4, and nucleosome disassembly^29,30^. Hyphal maintenance requires the recruitment of the Hda1 histone deacetylase by the transcription factor Brg1 to the promoters of hypha-specific genes. Hda1 deacetylates Yng2 (a subunit of the NuA4 histone acetyltransferase module), leading to reduction of chromatin H4 acetylation, nucleosome repositioning, and blockage of Nrg1 access to promoters of hypha-specific genes^29,30^. However, the regulatory link between NuA4 and Hda1 activity to nucleosome repositioning during hyphal elongation is yet to be uncovered.

In this study, we report a regulated merge and separation of the NuA4 and SWR1 complexes in *C. albicans* that controls the cell fate transition between the yeast and hyphal states. The merge is regulated by the acetylation of Eaf1 at K173 via the opposing actions of NuA4 and Hda1 at the promoter chromatin of hypha-specific genes. The Eaf1 K173 residue is conserved in Eaf1 orthologs of polymorphic fungi, but not conserved in Eaf1 orthologs from filamentous fungi or fungi of yeast form and higher eukaryotes. Our data suggest that the Eaf1 acetylation state at K173 at the promoter chromatin of hypha-specific genes is regulated by chromatin-associated NuA4 and Hda1 and controls the merge and separation of the SWR1 and NuA4 complexes and the morphological states.

## Results

### The Swr1 associates with Esa1 in yeast state and dissociates from Esa1 during hyphal elongation in *C. albicans*

*C*. *albicans* can transit dynamically between a unicellular yeast form and a multicellular hyphal form in response to nutritional and environmental cues that mimic the diverse microenvironments it encounters in its human host^28,31^. Temperature is one of the most important environmental cues. High temperature (37 °C) in combination with inoculation is sufficient for hyphal initiation. Hyphal elongation requires additional signals, such as serum or reduced Tor1 signaling^29^. Hyphal cells convert to yeast in rich media and the transition is sped up at low temperature (25 °C)^28^.

To understand the contributions of NuA4 and SWR1 to hyphal development, we first analyzed deletion mutants of the two core enzymes. The *swr1* mutant showed sustained hyphal elongation in rich media at 37 °C (Fig. 1b) and the phenotype is more obvious at 25 °C when wild-type (WT) hyphae cells started to grow yeast in 1 h (hr), whereas the mutant hyphae maintain its elongation state more than 12 h (Fig. 1c). This is in contrast to the *esa1* mutant, which was blocked for hyphal initiation (Fig. 1b)^32^.

Next, we determined whether there is any interaction between the two complexes during the reversible morphological transition. By tagging the two core enzymes, we observed that Swr1 and Esa1 were associated in yeast cells, but separated in hyphae (Fig. 1d). To further elucidate whether Swr1 and Esa1 complexes are associated together in yeast cells, we examined size/molecular weight (MW) of Swr1 and Esa1 complexes by gel filtration. Whole-cell extracts (WCEs) were prepared and subjected to gel filtration; Swr1 and Esa1 were detected by western blotting. In yeast state, Swr1 and Esa1 were present mainly in fractions eluting around 2 MDa that likely represents the merged complex of SWR1–NuA4 with predicted MW of 1.7 MDa (Fig. 1e, up panels). In contrast, in hyphae state, Swr1 and Esa1 were eluted at fractions around 1 MDa, which likely are separated SWR1 and NuA4 complexes with predicted MW around 0.9 MDa (Fig. 1e, down panels). It is interesting that predominately the merged complex was observed in yeast state, but not a mixture of both merged and separate complexes (Fig. 1e). Together, our data indicate that Swr1 and Esa1 are present in a larger complex in yeast cells. During hyphal development, Swr1 dissociated from Esa1 within 0.5 h of hyphal initiation and kept apart during hyphal elongation (37 °C, with serum) (Fig. 1f, left panel). In rich media (37 °C, without serum), the two enzymes dissociated around 0.5 h and re-associated by 2 h (Fig. 1f, middle panel). When hyphal cells were converted to yeast growth (25 °C, YPD), the two separated enzymes re-associated quickly and maintained the association in later points (Fig. 1f, right panel). Therefore, association and dissociation of Swr1 with Esa1 are correlated with the dynamic reversible morphological transitions.

### Yaf9 and Eaf1 bridge the SWR1–NuA4 complexes in yeast state

**Fig. 2:**
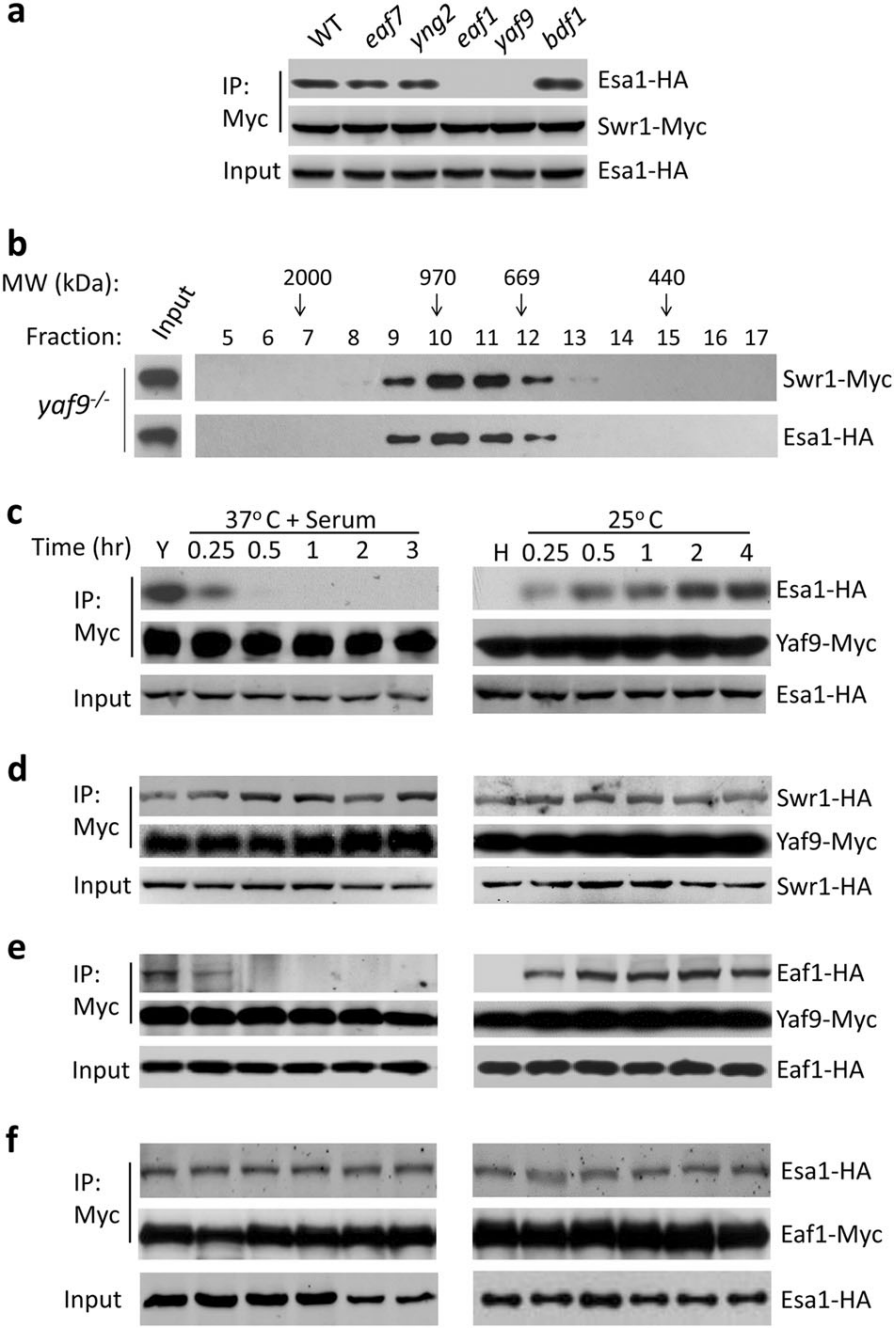
Yaf9 and Eaf1 bridge the SWR1 and NuA4 complexes. **a** Eaf1 and Yaf9 are required for the association between Esa1 and Swr1. Esa1-HA and Swr1-Myc were introduced into wild-type strain BWP17 or mutant strain (*eaf7*, *yng2*, *eaf1*, *yaf9*, and *bdf1*) respectively, and then cultured in yeast growth condition (YPD at 25 °C) for co-immunoprecipation (Co-IP) or immunoblotting (IB). **b** Gel filtration for the presence of Swr1 and Esa1 in yeast state of *yaf9* mutant. Same fractions eluted from a Superose 6 column were analyzed for Swr1-Myc or Esa1-HA by western blotting. **c** Yaf9-Esa1, **d** Yaf9-Swr1, **e** Yaf9-Eaf1, and **f** Esa1-Eaf1 association during the reversible yeast–hyphae transition. A wild-type strain (BWP17) harboring Yaf9-Myc together with HA-tagged Esa1, Swr1, or Eaf1 was cultured in YPD at 25 °C for 12 h (yeast state) or YPD with 10% serum at 37 °C for 3 h (hyphae state). The yeast cells were induced in YPD + 10% serum at 37 °C for hyphal development and the hyphae were re-cultured in fresh YPD medium at 25 °C for yeast growth. The cells collected at time points indicated, WCEs were subjected for Co-IP analysis

To determine which subunits of NuA4 and SWR1 mediate the Swr1–Esa1 association, we examined their interactions in mutants lacking subunit of NuA4 or SWR1 in yeast growth condition. The Swr1–Esa1 association was blocked in cells of *yaf9* or *eaf1* mutants, but was not affected in cells deleting other subunits, e.g., Eaf7, Yng2, or Bdf1 (Fig. 2a). Thus, Yaf9 and Eaf1 are required for mediating the Swr1–Esa1 association. We further examined the size of the Swr1 and Esa1 complexes in yeast cells of *yaf9* mutant by gel filtration. Both Swr1 and Esa1 were present in smaller size fractions around 1 MDa (Fig. 2b), confirming that Yaf9 is required for the formation of the merged Swr1–Esa1 complex in yeast.

To investigate how Yaf9 and Eaf1 mediate the Swr1–Esa1 association during reversible yeast–hyphae transition, we examined the interaction of Yaf9 with NuA4 or SWR1 complex represented by two core enzymes Esa1 or Swr1. Yaf9 associated with Esa1 in yeast state, dissociated rapidly during hyphal elongation (Fig. 2c, left panel), and re-associated with Esa1 when the hyphal cells converted to yeast growth (Fig. 2c, right panel). In contrast, Yaf9 associated constitutively with Swr1 in both yeast and hyphae (Fig. 2d). The data suggest that Yaf9 functions as a stable subunit of SWR1, but not of NuA4 in *C. albicans*. Consistently, the *yaf9*-null mutant phenocopyed the *swr1*-null mutant during yeast–hyphae transition, while both revertants exhibited WT phenotypes (Supplementary Fig. S1). This is different from *S. cerevisiae*, where Yaf9 is a stable subunit of both NuA4 and SWR1 complexes. We then analyzed the interaction between Yaf9 and Eaf1, a platform protein in NuA4. The Yaf9–Eaf1 interaction is regulated in a similar manner as the Yaf9–Esa1 interaction. The two proteins associated together in yeast state, departured from each other during hyphal development (Fig. 2e, left panel), and re-associated during hyphae-to-yeast conversion (Fig. 2e, right panel). As expected, the Eaf1 associated constitutively with Esa1 (Fig. 2f), regardless of morphological states. Our data suggest that Yaf9–Eaf1 interaction contributes to the integration of SWR1 and NuA4 complexes.

### YEATS domain of Yaf9 is important for Yaf9–Eaf1 interaction and hyphal elongation

**Fig. 3:**
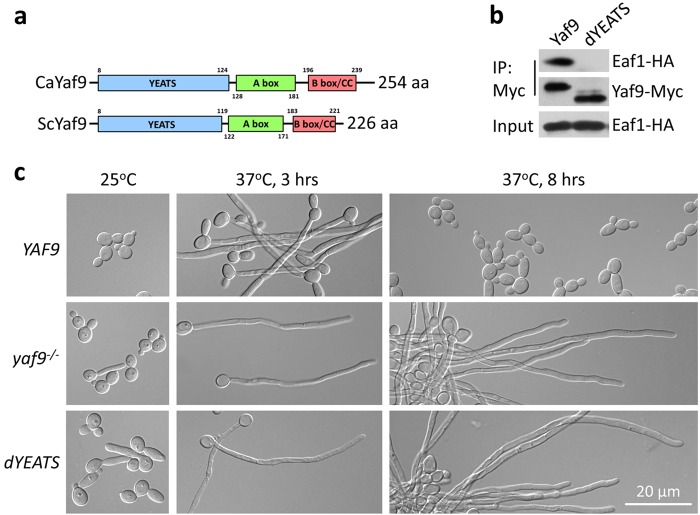
Yaf9 YEATS domain is critical for Yaf9–Eaf1 interaction. **a** Schematic comparison of *C. albicans* Yaf9 (CaYaf9) and *S. cerevisiae* Yaf9 (ScYaf9). **b** Interaction between Eaf1 and Yaf9 or Yaf9 without YEATS domain. Yaf9-Myc or Yaf9 deleting YEATS domain (Yaf9 dYEATS-Myc) was co-expressed with Eaf1-HA in wild-type strain (BWP17). The cell culture in YPD at 25 °C for 12 h were collected for Co-IP. **c** Cell morphology of wild-type *YAF9*, *yaf9* mutant, or *yaf9dYEATS* mutant. Cells were cultured in YPD at 25 °C for yeast growth or at 37 °C for hyphal elongation

*C*. *albicans* Yaf9 (CaYaf9) contains a conserved YEATS domain (Fig. 3a)^33^. YEATS domain in *S. cerevisiae* Yaf9 (ScYaf9) is structurally similar to histone chaperone Asf1 and is required for binding of Yaf9 to histones H3 and H4 in vitro^34^. To test whether the YEATS domain in *C. albicans* Yaf9 is responsible for binding to Eaf1, we deleted the YEATS domain in CaYaf9 and performed immunoprecipitation assay in vivo. As expected, a Yaf9 protein lacking the YEATS domain (Yaf9-dYEATS) failed to interact with Eaf1 in yeast cells (Fig. 3b). Thus, the YEATS domain of CaYaf9 is required to mediate the Yaf9-Eaf1 interaction. We then determined if the YEATS domain functions in hyphal elongation. The cells lacking full-length Yaf9 or the YEATS domain of Yaf9 showed sustained hyphal elongation at 37 °C without serum, a condition insufficient for sustained hyphal elongation (Fig. 3c). The phenotype is more obvious after longer incubation (37 °C, 8 h) when WT cells have converted back to yeast growth. Thus, the YEATS domain of Yaf9 is essential for the function of Yaf9 in *C. albicans*.

### Acetylation of Eaf1 at K173 has a key role in dynamic regulation of yeast–hyphae transition

**Fig. 4:**
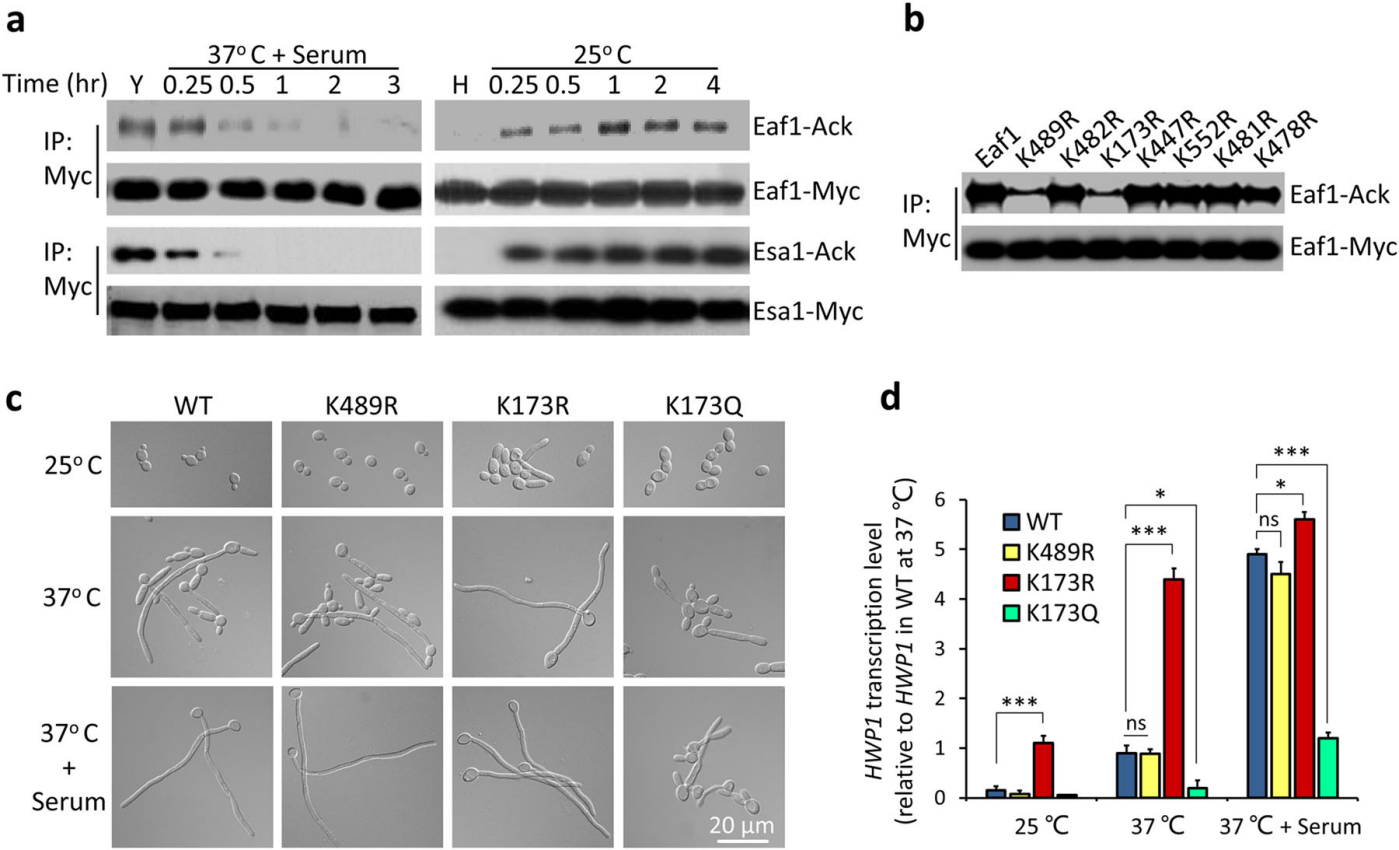
The regulated acetylation of Eaf1 at K173 has a key role in hyphal elongation. **a** Acetylation and deacetylation of Eaf1 and Esa1 during yeast–hyphae transition. Cells harboring Eaf1-Myc or Esa1-Myc were induced for hyphal development or for yeast conversion. WCEs were collected, immunoprecipitated with anti-Myc antibody, and probed with pan-lysine acetylation antibody. **b** Identification of acetylation sites in Eaf1. The pEaf1-myc and seven K to R mutants (pEaf1-myc muts) were introduced into wild-type strain (BWP17), respectively, and cultured in YPD at 25 °C, collected for acetylation detection. **c** Cell morphology of wild-type, *eaf1*^*K489R*^, *eaf1*^*K173R*^, and *eaf1*^*K173Q*^ mutants. Overnight cultured cells were inoculated into YPD and cultured at 25 °C for 6 h or at 37 °C with or without serum for 3 h. **d** Expression of hyphae-specific gene *HWP1* in *eaf1* mutants. The strains used in (**c**) were cultured at three growth conditions and collected for qRT-PCR. The signal obtained from *ACT1* mRNA was used as a loading control for normalization. The *HWP1* level in wild-type at 37 °C without serum is set to be 1.00. The data were presented as mean ± SEM, *n* = 3, *P < 0.05, **P < 0.01, ***P < 0.001

The YEATS domain in human AF9/Yaf9 was reported to recognize acetylated histone H3 and function as a reader^35^. Acetylation is also known to contribute to assembly of complex, such as TIP60–p400 complex^36^. It is possible that CaYaf9 recognizes acetylated Eaf1 to mediate the Yaf9-Eaf1 interaction. We approached this by first determining whether Eaf1 is acetylated. Using an anti-acetyl-lysine antibody, we found that Eaf1 was acetylated in the yeast cells, and the acetylation level decreased quickly during yeast to hyphae transition (Fig. 4a, left panel). Eaf1 was not acetylated in hyphae, but acetylated again during hypha-to-yeast transition (Fig. 4a, right panel). Similarly, the core enzyme Esa1 was also acetylated and deacetylated dynamically during the reversible yeast–hyphae transition, such as the Eaf1 (Fig. 4a). *C. albicans* Eaf1 has seven putative lysine acetylation sites K489, K482, K173, K447, K552, K481, and K478 (http://bdmpail.biocuckoo.org/). To determine which lysine is acetylated, we substituted each of the seven predicted lysine residues with arginine to mimic non-acetylated state and assayed acetylation level of Eaf1 in yeast growth conditions. Among the seven predicted lysine residues, K489 and K173 contributed to Eaf1 acetylation (Fig. 4b).

We further showed that only the mutation at K173 but not K489 affects hyphal elongation. The *eaf1*^*K489R*^ mutant had no defects in hyphal development (Fig. 4c) and expression of hypha-specific genes (Fig. 4d). However, the *eaf1*^*K173R*^ mutant (mimicking the constitutive deacetylation state) sustained hyphal elongation in YPD without serum at 37 °C (Fig. 4c). In contrast, the *eaf1*^*K173Q*^ mutant (mimic acetylation) could not maintain the hyphal elongation in serum containing media at 37 °C, although it could initiate hyphal development and form germ tube (Fig. 4c). Consistent with hyphal morphologies, the hyphae-specific gene *HWP1* was highly induced in *eaf1*^*K173R*^ cells and severely repressed in *eaf1*^*K173Q*^ cells (Fig. 4d). In a yeast growth condition (25 °C, YPD), the *HWP1* expression was detectable in *eaf1*^*K173R*^ cells, but not in the WT cells. In YPD media at 37 °C, *HWP1* was highly expressed in *eaf1*^*K173R*^ cells, but in low level in WT cells. In a hyphal development condition (37 °C, YPD plus serum), *HWP1* expression was highly induced in both WT and *eaf1*^*K173R*^ cells, but repressed in *eaf1*^*K173Q*^ cells (Fig. 4d). Interestingly, the *eaf1* deletion mutant was defective in hyphal elongation (Supplementary Fig. S2b), behaving similar to the Eaf1^K173Q^ mutant (Fig. 4c). However, the *eaf1*-null mutant had severe defects in cell growth, while the Eaf1^K173Q^ mutant was normal in cell growth (data not shown). Thus, the platform protein Eaf1 is likely required for the integrity of NuA4 complex, while the acetylation of Eaf1 at K173 mainly contributes to the regulation of dynamic yeast–hyphae transition.

### Yaf9 recognizes acetylated Eaf1 and mediates direct interaction between Yaf9 and Eaf1

**Fig. 5:**
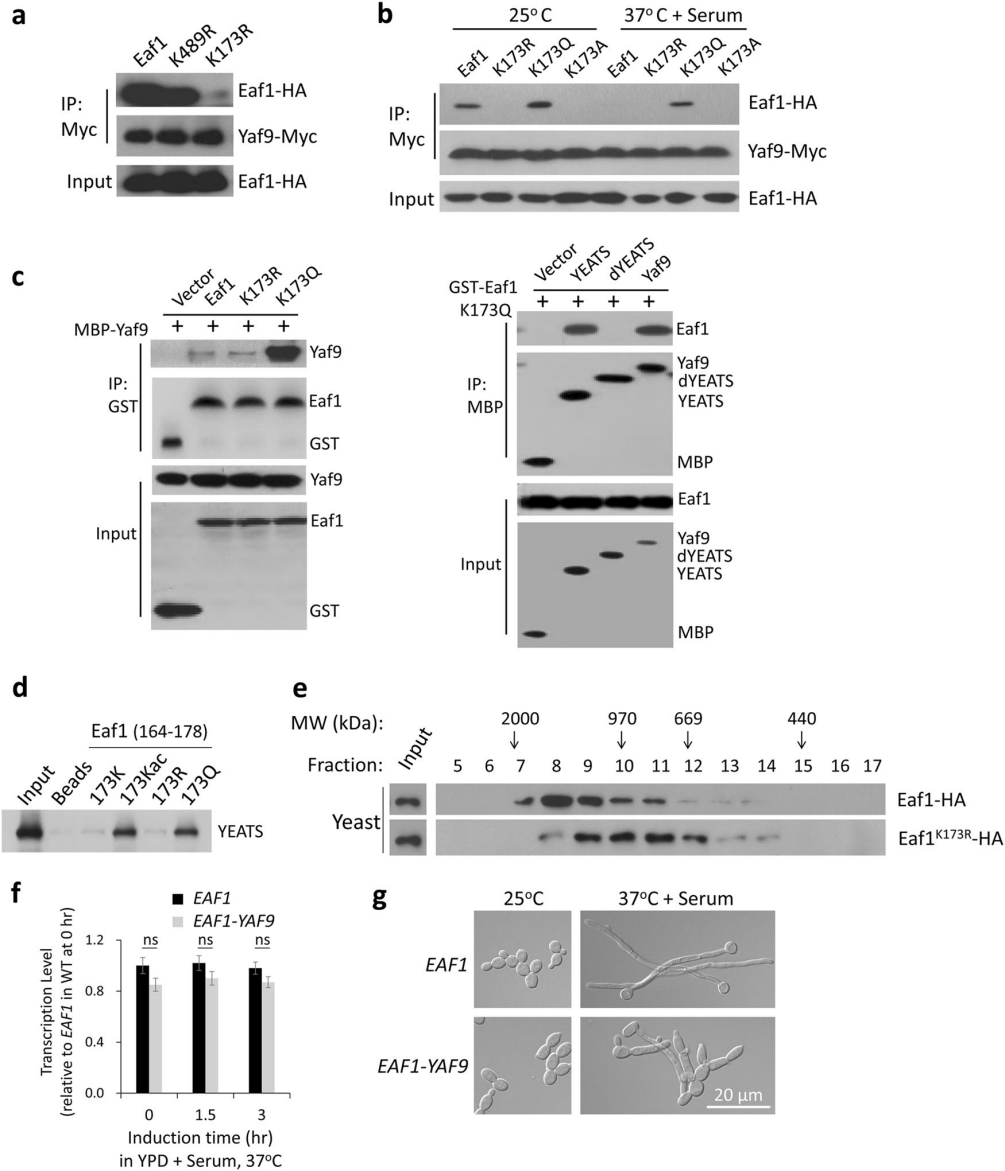
Yaf9 YEATS domain recognizes acetylated Eaf1 at K173. **a** Interaction of Yaf9 with Eaf1^K489R^ or Eaf1^K173R^. Yaf9-myc was co-expressed with HA-tagged wild-type Eaf1, Eaf1^K489R^, or Eaf1^K173R^ mutant in *C. albicans*. Yeast-state cells were used for Co-IP. **b** Effects of K to R, to Q, or to A mutation at residue 173 of Eaf1 on Yaf9–Eaf1 interaction. HA-tagged Eaf1, Eaf1^K173R^, Eaf1^K173Q^, or Eaf1^K173A^ was co-expressed with Yaf9-myc, and cultured in YPD at 25 °C or at 37 °C with serum for Co-IP. **c** Yaf9-Eaf1 interaction in vitro. *E. coli* purified MBP-tagged Yaf9 or Yaf9 mutants and GST-tagged Eaf1 or Eaf1 mutants were co-incubated at 4 °C for 2 h, and analyzed by Co-IP and IB. **d** Peptide pull-down assays using indicated Eaf1 (164–178 aa) peptides and MBP-tagged YEATS domain. **e** Gel filtration of WCEs. The yeast state cells carrying Eaf1-HA or Eaf1^K173R^-HA used in **b** were analyzed. The eluted fractions were immunoblotted with anti-HA antibody. **f** Expression of Eaf1–Yaf9 fusion. Overnight cultures of wild-type *EAF1* and *EAF1*-*YAF9* fusion were induced in YPD with 10% serum at 37 °C for indicated time and collected for qRT-PCR. The 0 h value of wild-type *EAF1* transcription is set to be 1.00. Error bars represent the SEM and ns means not significant, *n* = 3. **g** Cell morphology of Eaf1–Yaf9 fusion. Strains used in **f** were cultured in YPD at 25 °C for yeast growth or in YPD with 10% serum at 37 °C for hyphal development

To test whether the YEATS domain of *C. albicans* Yaf9 can directly recognize the acetylated Eaf1 and mediate the Yaf9–Eaf1 interaction, we performed immunoprecipitation assay both in vivo and in vitro. In yeast growth state, the K173R mutation reduced the level of their interaction, whereas the K489R mutation did not affect their interaction (Fig. 5a), indicating that acetylation at K173 but not K489 mediates the association between Eaf1 and Yaf9. Contrastingly, the K173Q mutation enhanced Eaf1–Yaf9 interaction in both yeast cells and hyphal cells (Fig. 5b). To test the charge effect on Yaf9–Eaf1 interaction, a K173A substitution to mimic charge neutralization created by acetylation reduced the interaction in both yeast and hyphae (Fig. 5b). Furthermore, another two mutants K173N (similar to Q) and K173H (positive charged) also reduced the interaction (Supplementary Fig. S2a). Consistently, the *eaf1*^*K173A*^, *eaf1*^*K173N*^, and *eaf1*^*K173H*^ mutants sustained hyphal elongation in YPD without serum at 37 °C (Supplementary Fig. S2b), phenocoping the *eaf1*^*K173R*^ mutant. Therefore, acetylation of Eaf1 at K173 is required for its recognition by Yaf9.

To test whether Yaf9 YEATS domain binds to Eaf1^K173Ac^ directly, we verified the interaction in vitro with recombinant proteins expressed and purified from *E. coli*. maltose binding protein (MBP)-tagged Yaf9 bound strongly to Eaf1^K173Q^, while less binding to Eaf1 or Eaf1^K173R^ (Fig. 5c, left panel). Conversely, GST-Eaf1^K173Q^ could bind to full-length Yaf9 or the YEATS domain, but not Yaf9 without the YEATS domain (Yaf9-dYEATS) (Fig. 5c, right panel). We further performed peptide pull-down assays by using four synthesized 15 aa peptides containing residue K173, K173ac, R173, and Q173, respectively. The K173ac- and Q173-containing peptides could pull down the MBP-tagged YEATS domain, while the K173 and R173 failed (Fig. 5d). Combining the in vivo and in vitro biochemical data, we suggest that the YEATS domain in *C. albicans* Yaf9 can recognize the acetylated Eaf1 at K173 to mediate their direct interaction. To test whether the single acetylated lysine at 173 can mediate the Yaf9–Eaf1 interaction and further bridge the merge of SWR1–NuA4 complexes, we next examined the sizes of NuA4 complex in WT Eaf1 or Eaf1^K173R^ mutant in yeast state by gel filtration. The Eaf1 was present in fractions eluting around 2 MDa corresponding to the larger complex, whereas the Eaf1^K173R^ mutant was only detected in smaller size fractions around 1 MDa (Fig. 5e), indicating that the acetylation of Eaf1 at K173 has a key role in controlling the merge of SWR1 and NuA4 complexes via Eaf1–Yaf9 interaction.

To further examine whether the Eaf1–Yaf9 interaction leads to yeast growth, we constructed a fusion protein, Eaf1–Yaf9, expressed under the endogenous *EAF1* promoter (Fig. 5f). Similar to Eaf1^K173Q^, the Eaf1–Yaf9 inhibited hyphal elongation and promoted hyphae-to-yeast transition (Fig. 5g). This demonstrated that constitutive interaction between Yaf9 and Eaf1 is inhibitory to hyphal elongation. The regulated dissociation between Yaf9 and Eaf1 during hyphal development by deacetylation of Eaf1 at K173 is critical for sustained hyphal development.

### Eaf1 is acetylated by Esa1/NuA4 and deacetylated by Hda1

**Fig. 6:**
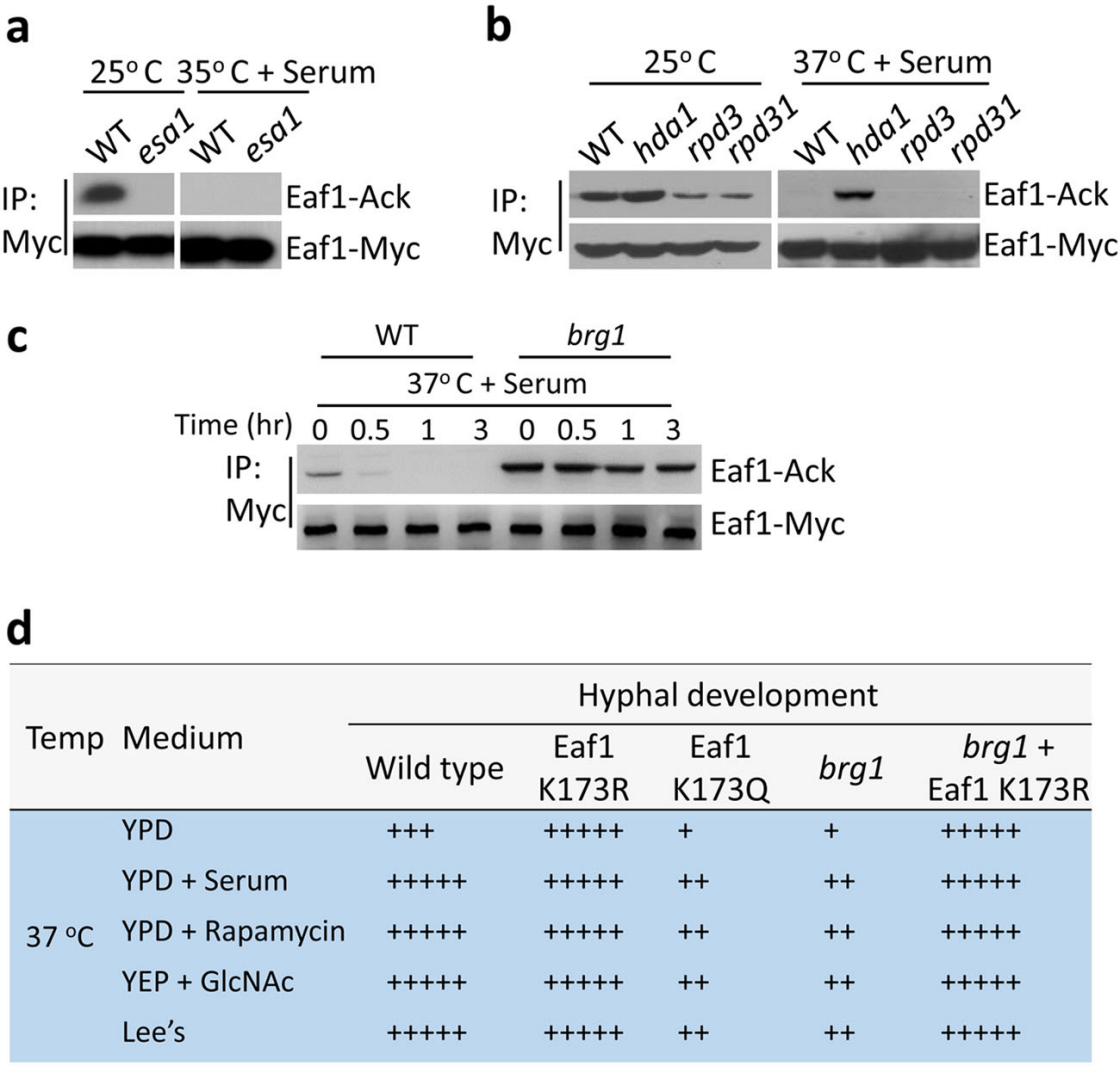
Hda1 and Esa1 regulate Eaf1 K173 acetylation via Brg1 recruitment. **a** Acetylation of Eaf1 in yeast cells requires Esa1. Eaf1-Myc was introduced into wild-type or *esa1* mutant cells and cultured in YPD at 25 °C for 6 h and in YPD plus 10% serum at 35 °C for 3 h. Then WCEs were collected, immunoprecipitated with anti-Myc, and probed with anti-Ac-lysine. **b** Deacetylation of Eaf1 in hyphal cells needs Hda1. Eaf1-Myc was introduced into wild-type, *hda1*, *rpd3*, and *rpd31* mutant, respectively, and cultured in YPD at 25 °C or YPD plus 10% serum at 37 °C for Co-IP and IB. **c** Eaf1 is deacetylated through a Brg1-dependent mechanism. Cells of wild-type or *brg1* mutant carrying Eaf1-myc were induced in YPD plus 10% serum at 37 °C and collected at time points indicated for Co-IP or IB. **d** Eaf1^K173R^ bypasses the requirement of Brg1 in hyphal elongation. Yeast cells of wild-type, Eaf1^K173R^, Eaf1^K173Q^, *brg1*, and *brg1* carrying Eaf1^K173R^ mutant were induced at 37 °C in YPD, YPD with 10% serum, YPD with 5 nM rapamycin, YEP with 2% GlcNAc, or Lee’s with mannitol for hyphal development. The level of hyphal elongation is indicated by the number of + symbols

To determine what regulates Eaf1 acetylation, we assayed its acetylation levels in mutants lacking the histone acetyltransferase, Esa1 of NuA4 complex^32^, or a histone deacetylase Hda1, Rpd3^37^ or Rpd31^38^. Eaf1 acetylation was abolished in *esa1*/*esa1*-null mutant cells in yeast growth condition (Fig. 6a), suggesting that NuA4 is required for the acetylation of Eaf1, the platform protein of NuA4. This is consistent with the functions of *S. cerevisiae* Esa1, which can acetylate a variety of non-histone substrates including subunits in NuA4 and Esa1 itself^6^. In *C. albicans*, Esa1 can also acetylate itself (Supplementary Fig. S3a and S3b) and Yng2 of the NuA4 complex^29^. The Eaf1 acetylation was abolished in the autoacetylation dead mutant Esa1^K296R^, but was strongly enhanced in the hyperactive mutant Esa1^K296Q^ (Supplementary Fig. S3c). The Esa1^K296R^ mutant blocked hyphal initiation, such as the *esa1*/*esa1*-null mutant, whereas the Esa1^K296Q^ mutant inhibited hyphal elongation (Supplementary Fig. S3d), phenocopying the *hda1*/*hda1*-null mutant and Eaf1^K173Q^ mutant. Therefore, Eaf1 is likely a direct substrate of Esa1. In three HDAC mutants lacking Hda1, Rpd3, or Rpd31 under hyphal induction conditions (37 °C with serum), the Eaf1 acetylation was only detected in the *hda1* cells, not in WT, *rpd3*, or *rpd31* mutants (Fig. 6b). Thus, deacetylation of Eaf1 in vivo depends on the Hda1 activity.

### Brg1 recruitment of Hda1 is required for deacetylation of Eaf1

*C. albicans* NuA4 complex is recruited to the promoters of hypha-specific genes and its HAT activity is required for hyphal initiation^32,39^. Hda1 is recruited to hypha-specific promoter by the transcription factor Brg1 and Hda1 activity is required for hyphal elongation^29,30,40^. To determine whether the deacetylation of Eaf1 via Hda1 is regulated in a chromatin-associated manner, we examined the acetylation level of Eaf1 in mutant cells lacking Brg1. Contrasting to WT cells in which Eaf1 is deacetylated quickly during yeast to hyphae transition, in *brg1*/*brg1* mutant cells the acetylation level of Eaf1 maintained at a high level during the entire hyphal development period (Fig. 6c). Therefore, deacetylation of Eaf1 during elongation is regulated by Brg1-recruited Hda1 at promoter chromatin. Together, our results suggest that Esa1- and Hda1-mediated acetylation and deacetylation of Eaf1 at K173 occur on chromatin.

### Eaf1^K173R^ bypassed the requirement of Brg1 in hyphal elongation

Many nutritional cues including serum, *N*-acetylglucosamine (GlcNAc), rapamycin, and Lee’s media can sustain hyphal elongation and they all require Brg1^29,30^. If Brg1 recruitement of Hda1 regulates Eaf1 K173 deacetylation and subsequent Swr1 dissociation, Eaf1 K173R (blocks acetylation) is expected to bypass the requirement of Brg1 or Hda1 and sustain hyphal development in the absence of the nutritional cues required for hyphal elongation, whereas Eaf1 K173Q (mimic acetylation) is expected to be defective in hyphal elongation despite the presence of the nutritional cues. Indeed, we find that Eaf1^K173R^ mutant cells sustained hyphal development in YPD (Fig. 6d and Supplementary Fig. S4), bypassing the requirement of the nutritional signals required for hyphal elongation, whereas Eaf1^K173Q^ mutant cells failed to sustain hyphae in all hyphal-inducing media tested (serum, GlcNAc, rapamycin, and Lee’s media) (Fig. 6d and Supplementary Fig. S4). Furthermore, the Eaf1–Yaf9 fusion cells exhibited similar defect in hyphal elongation as the Eaf1^K173Q^ mutant cells. Thus, the regulation of Eaf1–Yaf9 association via Eaf1 K173 acetylation bypassed the requirement of the nutritional and environmental signaling for hyphal elongation. To further demonstrate the epistatic relationship between Brg1/Hda1 and Eaf1 K173 acetylation in the regulation of hyphal elongation, Eaf1^K173R^ was transformed into the *brg1*/*brg1* deletion mutant. The *brg1*/*brg1* cells are defect in hyphal elongation in media containing serum, GlcNAc, rapamycin or in Lee’s media at 37 °C^30^. The *brg1*/*brg1* cells harboring the Eaf1^K173R^, however, formed true hyphae in all conditions at 37 °C (Fig. 6d and Supplementary Fig. S4). Thus, Eaf1^K173R^ bypassed the requirement of Brg1 in hyphal elongation. Our epistasis analysis demonstrates that chromatin recruitment of Hda1 by Brg1 is to deacetylate proteins in the NuA4 complex and release Swr1 for sustained hyphal elongation.

### Orchestrated promoter association of Esa1, Hda1, Swr1, and H2A.Z during the reversible yeast–hyphae transitions

**Fig. 7:**
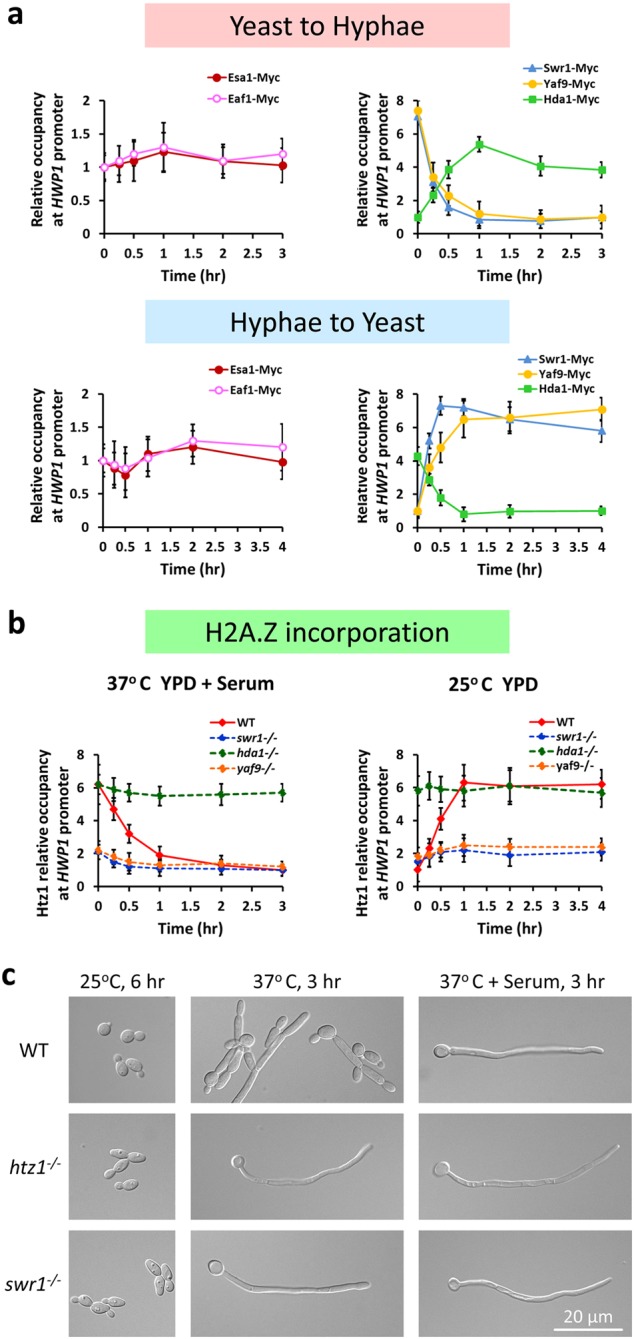
Association of NuA4–SWR1 proteins with the hypha-specific promoter during the reversible yeast–hyphae transitions. **a** Association of Myc-tagged Esa1, Hda1, Swr1, Eaf1, or Yaf9 with *HWP1* promoter during the reversible yeast–hyphae transitions by ChIP with anti-Myc antibodies. For yeast-to-hyphae transition, an overnight culture of wild-type strain carrying each Myc-tagged protein was diluted 1:100 into pre-warmed YPD at 37 °C in the presence of 10% serum and cells were collected at 0 min, 15 min, 30 min, 1 h, 2 h, and 3 h for ChIP experiments. ChIP DNA was quantitated by qPCR with primers at the UAS regions of *HWP1* as described^39^. The *ADE2* coding region was used as a control. The enrichment of each Myc-tagged protein is presented as a ratio of *HWP1* IP (bound/input) vs. the control IP (bound/input), the 0 h or 3 h value on *HWP1* was set to 1.00. For hyphae-to-yeast conversion, hyphae cells of each strain were cultured in YPD at 25 °C and collected at 0 min, 15 min, 30 min, 1 h, 2 h, and 4 h for ChIP-qPCR analysis; the 0 h or 4 h value on *HWP1* was set to 1.00. The ChIP data show the average of three independent qPCR data with error bars representing the SEM. **b** Deposition of Htz1 at the promoter of hypha-specific gene *HWP1*. Yeast state cells of wild-type, *swr1*, *yaf9*, and *hda1* mutant were induced in YPD plus 10% serum at 37 °C and hyphae state cells were cultured in YPD at 25 °C. ChIP-qPCR validation was determined using anti-Htz1 antibody. The 0 h value in the wild-type hyphae was set to be 1.00. **c** Cell morphology of wild-type, *htz1* or *swr1* mutant. Overnight cultured cells were inoculated into YPD medium and cultured at 25 °C for 6 h or at 37 °C with or without serum for 3 h

The coordinated activities of various chromatin remodeling and modifying complexes are crucial in maintaining promoter distinct chromatin to ensure appropriate gene expression. We performed chromatin immunoprecipitation (ChIP) analysis to determine the occupancy of Esa1, Hda1, and Swr1 on the promoter of the hypha-specific gene *HWP1*. During the reversible yeast–hyphae transition, Esa1 was associated with the *HWP1* promoter constitutively with a slight increase during the early stage of yeast-to-hyphae transition and a slight decrease during the early stage of hyphae-to-yeast conversion (Fig. 7a, left panels). In contrast, Hda1 association with the *HWP1* promoter was dynamically regulated during the yeast-to-hypha transition as reported^29^. The promoter-associated Hda1-Myc maintained in low level in yeast state, significantly increased during hyphal initiation, remained associated during hyphal elongation (Fig. 7a, right up) and decreased when hyphal cells were converted to yeast (Fig. 7a, right down). In contrast to Hda1, Swr1-Myc was highly associated with the *HWP1* promoter in yeast cells, dissociated quickly from the promoter during hyphal initiation, and remained unbound during hyphal development (Fig. 7a, right up). During hyphae-to-yeast transition, Swr1-Myc re-associated with the promoter right away and remained bound in later time points (Fig. 7a, right down). The promoter-associated Eaf1 and Yaf9 are also coordinately regulated. Eaf1 was associated with the *HWP1* promoter constitutively (Fig. 7a, left panels), like the Esa1, suggesting these two components of NuA4 complex work together on chromatin. As a component of SWR1 complex, Yaf9 association with the *HWP1* promoter is similar to the Swr1, which was dynamically regulated during the yeast-to-hypha transition (Fig. 7a, right panels). Therefore, during the reversible yeast–hyphae transitions, Hda1-myc and Swr1-myc demonstrated opposite patterns of promoter association, consistent with Hda1-regulated dissociation of Swr1 from the chromatin-bound NuA4 via deacetylation of Eaf1.

SWR1 is the principal complex responsible for the incorporation of H2A.Z into chromatin, and NuA4 HAT activity appears important for the association of H2A.Z and SWR1 with chromatin in *S. cerevisiae*^41–44^. Crosstalk between SWR1 and NuA4 facilitates the H2A.Z deposition and modification, the shared subunits functioning as a docking platform to facilitate the exchange of unique NuA4 and SWR1 subunits on chromatin^5^. To understand the contributions of NuA4 and SWR1 in the incorporation of H2A.Z during reversible yeast–hypha transitions in *C. albicans*, we performed ChIP analysis to examine the deposition of H2A.Z (Htz1) at the promoter of hypha-specific gene *HWP1*. In WT strain, the promoter-associated Htz1 was dynamically regulated during the reversible yeast–hypha transition (Fig. 7b). Similar to Swr1, Htz1 was highly associated with the *HWP1* promoter in yeast cells, dissociated quickly from the promoter during hyphal initiation, and remained unbound during hyphal elongation (Fig. 7b, left panel). When hyphal cells were converted to yeast, Htz1 re-associated with the promoter right away and remained bound (Fig. 7b, right panel). In contrast, the regulated patterns of promoter association were all abolished in *swr1*, *yaf9*, or *hda1* mutant (Fig. 7b). The promoter-associated Htz1 maintained in low level in *swr1* or *yaf9* mutant, but in high level in *hda1* mutant. Thus, Hda1-regulated dissociation of SWR1 prevented the incorporation of H2A.Z into chromatin at hypha-specific promoter.

We further analyzed the role of H2A.Z in hyphal development. The *htz1* mutant showed sustained hyphal elongation in rich media at 37 °C (Fig. 7c), phenocopying the *swr1* mutant or *yaf9* mutant. Together, our data suggest that the incorporation of H2A.Z into chromatin depends on SWR1, which is merged with NuA4 in yeast state. During hyphal elongation Hda1-regulated dissociation of SWR1 from the NuA4 prevents incorporation of H2A.Z into the chromatin around Hda1.

## Discussion

### Acetylation of Eaf1 K173 controls merge of NuA4–SWR1 complexes and yeast–hypha transitions in *C. albicans*

**Fig. 8:**
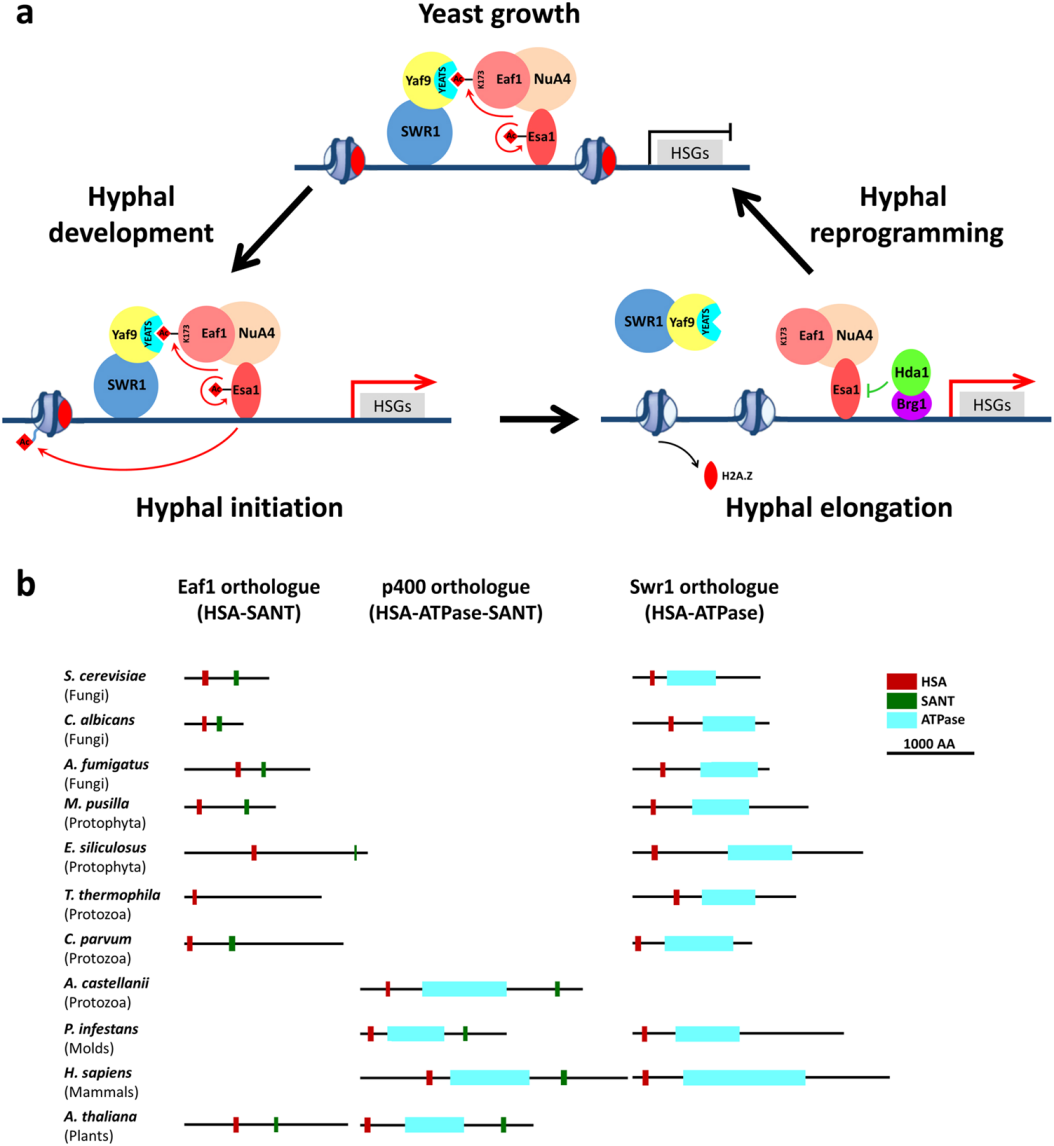
Model for dynamic merge and separation of NuA4 and SWR1 complexes. **a** Model for dynamic merge and separation of NuA4 and SWR1 complexes during reversible yeast–hypha transition of *C. albicans*. In yeast growth state, NuA4 and SWR1 merge together via the Eaf1–Yaf9 interaction. During hyphal development, NuA4–SWR1 is un-separated at the early stage, but separates into two distinct complexes during hyphal elongation. When hyphae convert to yeast, the two complexes remerge into one complex. The reversible acetylation and deacetylation of Eaf1 at K173 by the NuA4 core enzyme Esa1 and the histone deacetylase Hda1 control the merge and separation of NuA4 and SWR1, and this regulation is triggered by Brg1 recruitment of Hda1 to chromatin in response to nutritional signals that sustain hyphal elongation and hypha-gene expression. Red arrows represent acetylation is activated and green lines represent acetylation is inhibited. The expression of hypha-specific genes (HSGs) is activated in hyphae state, but repressed in yeast state. **b** Predicted orthologues of Eaf1, p400 and Swr1 in eukaryotes. Protein domains HSA, ATPase, and SANT form three different domain architectures, which occur in various combinations across eukaryotes. In fungi, each genome contains an Eaf1 ortholog and a Swr1 ortholog, but no p400-like proteins. In protista, Eaf1 and Swr1 orthologs are found in some genomes of protophyta or protozoa, p400-like proteins, as well in protozoa. The p400-like proteins are broadly found in higher eukaryotes of both animalia and plantae. In addition of a p400-like protein, a Swr1-like protein is found in genome of metazoa, and an Eaf1-like protein in plants. The orthologs of Eaf1, p400, and Swr1 are analyzed in each sequenced genome (http://blast.ncbi.nlm.nih.gov/Blast.cgi)

In this study, we demonstrate a dynamic merge and separation of NuA4 and SWR1 complexes during transition between yeast growth and hyphal development in *C. albicans*. In yeast growth state, NuA4 and SWR1 merge together via the Eaf1–Yaf9 interaction, such as the large complex TIP60 in human. During hyphal development, NuA4 and SWR1 separate into two distinct complexes such as their homologs in *S. cerevisiae*. When hyphae convert to yeast, the two complexes remerge into one complex (Fig. 8a). We show that the dynamic merge and separation of NuA4 and SWR1 are critical in regulating hyphal development and cell fate plasticity. This is the first discovery of a regulated merge and separation of the NuA4 and SWR1 complexes during development and the regulation is critical for cell fate determination.

We demonstrate that acetylation of Eaf1 K173 controls the merge between the NuA4 and SWR1 complexes. The acetylation and deacetylation of Eaf1 at K173 are regulated by the NuA4 core enzyme Esa1 and the histone deacetylase Hda1, respectively. In our previous work, we demonstrated that NuA4 Esa1 activity is essential for hyphal initiation^32^. The transcription factor Efg1 recruited NuA4 complex activates the nucleosomal H4 acetylation at hypha-specific promoters during hyphal initiation^39^. During hyphal elongation, Brg1 recruitment of Hda1 to promoter chromatin deacetylates Esa1, Yng2, and Eaf1 to downregulate NuA4 activity^29^ (Supplementary Fig. S3). NuA4 Esa1 activity is essential for hyphal initiation, but is inhibitory for hyphal elongation^29,32,39^. Therefore, Brg1-recruited Hda1 on promoter chromatin deacetylates Eaf1, Yng2, and Esa1; the deacetylated and inactivated Esa1 in turn prevents the re-acetylation of Eaf1 during hyphal development. When hyphae convert to yeast growth, Brg1/Hda1 de-associate from chromatin and the Esa1 is auto-acetylated and activated. Thus, the reversible acetylation and deacetylation of Eaf1 by Esa1 and Hda1 control the merge and separation of NuA4 and SWR1 precisely, and this regulation is triggered by Brg1 recruitment of Hda1 to chromatin in response to nutritional signals that sustain hyphal elongation. This study uncovers the molecular mechanism underlying the NuA4/Hda1 controlled chromatin remodeling during hyphal elongation. The incorporation of H2A.Z into chromatin depends on SWR1 and is orchestrated during the reversible yeast–hypha transitions in *C. albicans*. In yeast state, SWR1 and NuA4 merge together and facilitate the H2A.Z deposition; during hyphal initiation, crosstalk between SWR1 and NuA4 facilitates the H2A.Z modification; during hyphal elongation, Hda1-regulated dissociation of SWR1 prevents the incorporation of H2A.Z and results in the eviction of H2A.Z from chromatin around Hda1. During hyphal reprogramming, when hyphae convert to yeast growth, the remerged complexes promote the incorporation of H2A.Z into chromatin at hypha-specific promoter.

*C. albicans* Yaf9 bridges the NuA4 and SWR1 complexes via anchoring to the acetylated Eaf1. Unlike *S. cerevisiae* Yaf9, which is one of the four shared components of NuA4 and SWR1^18^, *C. albicans* Yaf9 is found to be a stable subunit of SWR1 but is only associated with NuA4 when Eaf1 of NuA4 is acetylated. Yaf9 consistently binds with Swr1, the platform protein of SWR1, and anchors to the acetylated Eaf1, the platform protein of NuA4, but separates from deacetylated Eaf1. Auger et al.^15^ proposed that Eaf1 and Swr1 are combined into a single large p400 protein, which led to the fusion of NuA4 and SWR1 in higher eukaryotes. We are the first to show that Yaf9 mediates a regulated association of Eaf1 and Swr1 to mimic the p400 in human TIP60 during cell fate differentiation, resulting in the fate-specific merge of NuA4 and SWR1. Noteworthy, *C. albicans* Yaf9 contains a YEATS domain that is found in many important chromatin-modifying and transcription complexes, forms a potential acetyl-lysine binding pocket and functions as a reader to recognize acetylated histone H3^34,35,45,46^. We demonstrate here that the YEATS domain of Yaf9 in *C. albicans* can recognize an acetyl-lysine of non-histone protein Eaf1.

### Evolutionary diversity in merged NuA4–SWR1 complex in eukaryotes

NuA4 and SWR1 are highly conserved from yeast to human. Some evidence suggests that these two complexes merged together since the origin of metazoan^5^. The platform protein Eaf1 of *S. cerevisiae* NuA4 complex contains an HSA domain and a highly positive-charged SANT domain. The platform protein Swr1 of *S. cerevisiae* SWR1 complex contains an HSA domain and ATPase domain. In comparison, the platform protein p400 of human NuA4 complex (TIP60) contains an HSA domain, ATPase domain, and SANT domain, which is proposed to act like a large platform protein of merged Eaf1 and Swr1 (Fig. 8b).

In fungi, homologs of NuA4 and SWR1 subunits have been predicted in each sequenced genome (http://blast.ncbi.nlm.nih.gov/Blast.cgi). The acetyltransferase Esa1 in *S. cerevisiae* is essential for general cell growth^11,12^. The *C. albicans* Esa1 is not essential for general cell growth, but is essential for hyphal development^32^. The *Aspergillus nidulans* Esa1, EsaA is found important for activation of secondary metabolite production^47^. The *C. albicans* Swr1 has a role in nucleosome positioning and is required for stabilization of a repressive chromatin state during white to opaque switching^48^. Both Eaf1 ortholog and Swr1 ortholog are found in each sequenced fungal genome, whereas p400-like proteins are absent, reflecting the existence of two distinct complexes, NuA4 and SWR1 (Fig. 8b).

In protista, homologs of Eaf1 and Swr1 have been predicted in genomes of some protophyta species including green algae and brown algae, but are not found in red algae and dinoflagellates (http://blast.ncbi.nlm.nih.gov/Blast.cgi). The Eaf1 and Swr1 homologs have also been predicted in protozoa species including ciliates, sporozoans and amoeboids, but not in flagellates (Fig. 8b). Surprisingly, a predicted p400-like protein is found in *Acanthamoeba castellanii*, which belongs to amoeboids (Fig. 8b), suggesting that merger of the two complexes probably has occurred in some protozoa. Interestingly, in addition to a Swr1-like protein, a p400 ortholog is predicted in molds, a fungus-like organism, reflecting merger of the two complexes in lower eukaryotes (Fig. 8b). The p400-like proteins are broadly found in higher eukaryotes of both animalia and plantae^49^. In addition of the p400-like proteins, Swr1-like proteins are found in metazoa and Eaf1-like proteins in plants^49^, suggesting the existence of a larger SWR1 complex (TIP60-like) and a smaller SWR1 complex in metazoan, and a larger NuA4 complex (TIP60-like) and a smaller NuA4 complex in plant (Fig. 8b).

### The acetylable lysine of Eaf1 provides a state for regulated dissociation of SWR1 from NuA4 and chromatin to control the transcription program of cell fate genes

The acetylable residue K173 is conserved in Eaf1 orthologs among polymorphic fungi, but is occupied by a non-acetylable residue arginine (R) in non-dimorphic yeast-like fungi or glutamine (Q) in filamentous fungi. Interestingly, the lysine residue is neither conserved among Eaf1 orthologs from protista, nor the Eaf1 orthologs from higher eukaryotes or p400-like proteins (Supplementary Fig. S5).

The acetyl-lysine at residue 173 endows Eaf1 the ability to regulate the *C. albicans* switching between yeast and hyphae quickly and reversibly. Both K to R and K to Q mutations of Eaf1 lead to the loss its regulatory ability on fast dynamic morphological transition in *C. albicans*. The non-acetylable arginine (R) in Eaf1 of *S. cerevisiae* may correlate with its non-dimorphic yeast phenotype, and smooth yeast-like phenotype of *Candida glabrata*. The glutamine (Q) residue mimicking constitutive-acetylation state in Eaf1 of *Aspergillus fumigatus* and *Fusarium graminearum* may correlate with their constitutive filamentous phenotype. The acetyl-lysine of Eaf1 may be evolutionarily important for fast dimorphic transition of fungi in response to multiple environmental stimuli. The non-modifiable residues including arginine and glutamine are unable to regulate the rapid reversible morphological transitions. The acetylable lysine at 173 may provide a state for Eaf1 to regulate association or dissociation between NuA4 and SWR1, which in turn controls the chromatin of fate-specific genes for activation or repression during cellular reprogramming.

The lysine residue seems not conserved in the Eaf1 orthologs from protista, which is probably correlated with their slow-morphological changing phenotype. The lysine residue is also not conserved in p400-like proteins from higher eukaryotes, as NuA4 and SWR1 have been evolutionarily merged into a larger TIP60-like complex by integrating Eaf1 and Swr1 into p400, and regulated merge and separation of NuA4 and SWR1 complexes are no longer necessary for a constitutive complex (Supplementary Fig. S5). In support of this prediction, all five acetylated lysine residues identified in human p400 are not located at regions conserved for acetyl-lysine of the Eaf1^36^. This layer of regulation seems to be evolutionarily discarded in higher eukaryotes. Thus, the Eaf1 K173 acetylation is a special layer of regulation for rapid dynamic reversible morphological transition in fungal kingdom. *C. albicans* is a human commensal fungus, which possesses the unique ability to achieve rapid and reversible cell fate changing between yeast and hyphae in response to various host niches. During its evolution as a commensal organism in the human host, *C. albicans* may have developed an elaborate mechanism to regulate its morphogenetic programs in order to survive in the host. Our results demonstrate a novel mechanism for dynamic merge and separation of NuA4 and SWR1 complexes during cell fate change and add a new layer for regulation of cell fate reprogramming.

## Materials and methods

### Media and growth conditions

*C. albicans* strains were routinely grown at 30 °C in YPD (1% yeast extract, 2% peptone, 2% dextrose). Transformants were selected on synthetic medium (2% dextrose, 0.17% Difco yeast nitrogen base w/o ammonium sulfate, 0.5% ammonium sulfate and auxotrophic supplements). For yeast growth, overnight culture was re-inoculated to fresh YPD medium and cultured at 25 °C for 6 h. For hyphal development, overnight cultured yeast cells were induced in media and conditions described previously^29,30,32^. For reversing hyphae to yeast, hyphae cells induced in YPD + 10% serum for 3 h at 37 °C, then transferred to fresh YPD medium and further cultured at 25 °C. The time course of yeast-to-hyphae or hyphae-to-yeast was listed in each figure or figure legend.

### Plasmid and strain construction

The *C. albicans* strains used in this study are listed in Table S1. Plasmids are listed in Table S2. Primers are listed in Table S3. For knocking out *C. albicans* genes, a combined PCR based method and URA BLAST method was used to delete first copy and second copy of the target genes, including *EAF1*, *YAF9*, *SWR1*, *BDF1*, and *HTZ1*. The detailed method has been previously described^32^. The revertants were constructed by re-introducing the genes into the knockout strains to confirm the phenotype of knocking out. The strains of the Swr1 revertant, Yaf9 revertant (Supplementary Fig. S1), and Eaf1 revertant (Supplementary Fig. S2) exhibited like WT strain during hyphal development. To construct the single amino acid mutant strains, the first copy of the gene was deleted and the second copy was replaced with the single residue mutant. To construct the plasmids for endogenous expression of Myc-tagged protein in *C. albicans*, the PCR fragment corresponding to Yaf9, Swr1 and Eaf1 respectively, were inserted into the BamHI–MluI sites of pPR673. The resulting plasmids were digested with SacI and integrated into their own loci for expression of Yaf9-Myc, Swr1-Myc and Eaf1-Myc in *C. albicans* cells. To construct the plasmids for endogenous expression of HA-tagged protein in *C. albicans*, the PCR fragment corresponding to Esa1, Swr1, Hda1, Rpd3 and Rpd31 respectively, were fused by Chang’s PCR strategy^50^. The PCR products of YAF9 and YAF9-dYEATS were inserted into the XbaI–MluI sites of pBES116. The resulting plasmids were digested with SacI and integrated into their own loci for expression of Yaf9 and Yaf9-dYEATS in *C. albicans* cells.

### Gel filtration

Gel filtration experiments were performed in a Superose 6 10/300 GL column (GE Healthcare) using an ÄKTAFPLC system. Yeast-state or hyphae-state cells were collected and washed once with phosphate-buffered saline (PBS) and then re-suspended in lysis buffer (25 mM Tris-HCl pH 7.5, 100 mM NaCl, 1 mM dithiothreitol (DTT), 1 mM phenylmethylsulfonyl fluoride (PMSF), protease inhibitor cocktail). The cells were homogenized by a high-pressure homogenizer (EmulsiFlex-C5, Avestin, Inc., Ottawa, Canada), then were centrifuged at 13,000 r.p.m. for 20 min. The supernatant was filtrated with a 0.22 μm membrane, 500 μL of WCEs (5 mg total protein) were injected into Superose 6 column, eluted at a speed of 300 μL/min. The eluted fractions were collected and concentrated by centrifugal filter devices (Amicon Ultra-4, 10 K, Millipore) and further analyzed by western blotting.

### Quantitative RT-PCR

Methods for RNA isolation were carried out as previously described^32^. For quantitative real-time reverse-transcription PCR analysis, 10 µg of total RNA was DNase-treated at 37 °C for 1 h using the DNA-free kit (Qiagen), cDNA was synthesized using the SuperScript II Reverse Transcriptase kit (Invitrogen), and quantitative PCR (qPCR) was performed using the iQ SYBR Green Supermix (Bio-Rad) with primers for *HWP1* and *ACT1*. The iCycler iQ detection system (Bio-Rad) was used for PCR amplification. Amplification specificity was determined by melting curve analysis.

### Bacterial expression and protein purification

*EAF1* was cloned into pGEX-4T1 with an N-terminal GST tag and *YAF9* was cloned into pSJ8 with an N-terminal MBP tag (Novagen). The Eaf1 mutants were constructed using the QuikChange Site-Directed Mutagenesis kit (Strategene). The recombinant proteins were expressed in *Escherichia coli* BL21 (DE3) Codon-Plus strain (Novagen). The cells were grown at 37 °C in LB medium containing 0.05 mg/mL ampicillin or kanamycin to log phase and induced with 0.25 mM IPTG at 16 °C for 24 h. The cells were collected and sonicated in a lysis buffer (20 mM Tris-HCl pH 8.0, 1 mM MgCl_2_, 150 mM NaCl, and 1 mM PMSF) and then centrifuged at 40,000 × *g* for 40 min. Eaf1 or Yaf9 was purified separately by affinity chromatography using a GST column (GE Healthcare) or a MBP column, respectively.

### In vitro GST and MBP pull-down

To detect the interaction between Eaf1 and Yaf9 in vitro, GST-Eaf1 (150 μg) and MBP-Yaf9 (100 μg) were incubated at 4 °C for 2 h within 1 mL lysis buffer supplemented with 1 mM GTP and 1 mM DTT. To pull down Yaf9 and Yaf9 mutants, GST-Eaf1 was purified by GST affinity chromatography on glutathione-coated Sepharose beads (GE Healthcare) in the lysis buffer, as recommended by the manufacturer. To pull down Eaf1 and Eaf1 mutants, MBP-Yaf9 was purified by MBP affinity chromatography on glutathione-coated Sepharose beads (GE Healthcare) in the lysis buffer. The beads were washed three times (10 min each) with the same buffer and then analyzed by co-immunoprecipitation (Co-IP) or IB with anti-GST antibody (Sigma) or anti-MBP antibody (NEB). The pGEX-4T1 or pSJ8 vector was used as control.

### Peptide pull-down assay

Two micrograms of biotinylated Eaf1 peptides (164–178 aa) with different residues at the 173 were incubated with 2 μg of MBP-tagged YEATS domain in binding buffer (50 mM Tris-HCl, pH 7.5, 200 mM NaCl, 0.1% NP-40, 1 mM PMSF) at 4 °C for overnight with rotation. Five microliters of Streptavidin beads (Amersham) were added to the mixture, followed by 1 h incubation with rotation. Then the beads were washed three times and the proteins were analyzed by western blotting.

### Immunoprecipitation

To detect the interaction between proteins in vivo, Co-IP analysis were carried out as previously described^39^. *C. albicans* cells carring Myc or HA-tagged proteins were collected by centrifuged at 5,000 r.p.m. for 5 min and washed with cold PBS twice. The cells were re-suspended in lysis buffer (50 mM HEPES pH 7.5, 140 mM NaCl, 1 mM EDTA, 1% Triton X-100, 0.1% sodium deoxycholate with protease inhibitor cocktail) and homogenized with acid-washed glass beads (Sigma) by a Fast-Prep system (FP120; Thermo Electron, Waltham, MA), and then centrifuged at 13,000 r.p.m. for 15 min. Lysates were immunoprecipitated with protein A-sepharose beads (GE Healthcare) conjugated with anti-Myc or anti-HA antibody (Santa Cruz) at 4 °C for 4 h. The immunoprecipitated complex was centrifuged, washed with lysis buffer, and detected with antibodies.

For testing the acetylation of Esa1 and Eaf1, Esa1-myc- and Eaf1-Myc-containing cells were cultured and collected, washed twice by cold PBS, and then re-suspended in 800 µl of 4 °C lysis buffer (50 mM HEPES pH 7.5,140 mM NaCl, 1 mM EDTA, 1% Triton X-100, 0.1% sodium deoxycholate) with protease inhibitors and 10 μM TrichostatinA (Sigma). Lysates were immunoprecipitated with 30 µL protein A-sepharose beads (GE Healthcare, 17–0974) conjugated with 5 µL of Myc antibody (Santa Cruz) at 4 °C for 4 h. The immunoprecipitated complex was washed by Lysis buffer (must add TrichostatinA all the time) and detected with a peroxidase-conjugated rabbit polyclonal anti-acetylated-lysine (Cell Signaling, 9441 S) antibody (Roche).

### Chromatin immunoprecipitation

ChIP assays were performed as described previously with some modification^29,39^. Briefly, *C. albicans* cells were cross-linked with 1% formaldehyde and suspended in lysis buffer (50 mM HEPES pH 7.5, 150 mM NaCl, 1 mM EDTA, 1% Triton X-100, 0.1% Na-Deoxycholate, 1 mM PMSF, protease inhibitors cocktail). Cells were lysed using glass beads and were sonicated to shear the chromatin to fragment sizes of ∼200–500 base pairs. Cross-linked chromatin fragment were immunoprecipitated with antibodies that specifically recognized Myc or Htz1 (Millipore # 07–718). Protein A/G-Sepharose beads (GE) were then added into the samples and the immunoprecipitated complexes were washed gradually with lysis buffer with a higher NaCl concentration of 500 mM, wash buffer (50 mM HEPES pH 7.9, 300 mM NaCl, 1 mM EDTA, 1% Triton X-100, 0.5% NP-40, 0.1% Na-Deoxycholate), and TE buffer (10 mM Tris HCl pH 8.0, 1 mM EDTA). Next, the immunoprecipitated complexes were eluted from beads with elution buffer (10 mM Tris-HCl pH 8.0, 1 mM EDTA, 1% SDS). Formaldehyde cross-linking was reversed by incubating the eluates at 70 °C 3 h. Eluted DNA was treated with 100 µg/ml proteinase K and purified with QIAquick PCR purification Kit (Qiagen). Immunoprecipitated fractions and WCEs containing DNA were analyzed by PCR. qPCR was performed with Power SYBR Green PCR Master Mix (Applied Biosystems).

### Statistical analysis

The data was presented as mean ± SEM. A two-tailed unpaired Student’s *t*-test was performed to compare the differences between treated groups relative to their paired controls. *p* < 0.05 was considered significant; *p < 0.05; **p < 0.01; ***p < 0.001. Statistical analyses were performed using GraphPad Prism Version 5.01 (GraphPad Software, Inc.)

## Electronic supplementary material

Supplementary Information

## Electronic supplementary material

**Supplementary Information** accompanies the paper at (10.1038/s41421-018-0043-0).
