# Supplementary material for: Merge and separation of NuA4 and SWR1 complexes control cell fate plasticity in Candida albicans
Source: Cell Discov. 2018 Aug 14;4:45. doi: 10.1038/s41421-018-0043-0 (PMC6089883; doi:10.1038/s41421-018-0043-0)
Supplement: Supplementary file 1 — Supplementary Information [file 41421_2018_43_MOESM1_ESM.pdf]

## Supplementary materials

**Table S1. *Candida albicans* strains used in this study**

**Table S2. Plasmids used in this study**

**Table S3. Primers used in this study**

**Figure S1. Cell morphology of *yaf9* or *swr1* mutants and revertants.** The strains were cultured overnight at 25 °C in YPD (up panels), and induced in YPD (middle panels) or YPD with 10% serum (down panels) at 37 °C for 3 hrs. Cell-morpha were observed and photographed.

**Figure S2. Functional analysis of Eaf1 K173 mutants. (a)** Effects of K to R, to Q, to N, or to H mutation at residue 173 of Eaf1 on Yaf9-Eaf1 interaction. HA tagged Eaf1, Eaf1$^{K173R}$, Eaf1K$^{173Q}$, Eaf1$^{K173N}$ or Eaf1$^{K173H}$ was co-expressed with Yaf9-myc, and cultured in YPD at 25 °C for Co-IP. **(b)** Cell morphology of wild-type, *eaf1$^{K173A}$*, *eaf1$^{K173N}$*, *eaf1$^{K173H}$*, *eaf1* mutants and *eaf1* revertant. Overnight cultured cells were inoculated into YPD and cultured at 37 °C with or without serum for 3 hrs.

**Figure S3. The role of auto-acetylation of Esa1 during hyphal development. (a)** Sequence comparison of regions around auto-acetylated lysine residue of *C. albicans* Esa1 (CaEsa1) and *S. cerevisiae* Esa1 (ScEsa1). **(b)** CaEsa1 is auto-acetylated at lysine 296. Cells carring Esa1-Myc or Esa1$^{K296R}$-Myc were cultured in YPD at 25 °C and the WCEs were collected, immunoprecipitated with anti-Myc, and probed with anti-Ac-K. **(c)** The Eaf1 acetylation was abolished in *esa1$^{K296R}$* mutant, but enhanced in *esa1$^{K296Q}$* mutant. Eaf1-Myc was introduced into cells of wild-type, *esa1*, *esa1$^{K296R}$*, and *esa1$^{K296Q}$* mutant, cultured in YPD at 25 °C or in YPD plus 10% serum at 35 °C for Co-IP or IB. **(d)** The Esa1$^{K296R}$ mutant blocks hyphal initiation, while the Esa1$^{K296Q}$ mutant inhibits hyphal elongation. The cells of wild-type, *esa1*, *esa1$^{K296R}$*, *esa1$^{K296Q}$*, and *hda1* mutant were cultured in YPD at 25 °C for 6 hrs or in YPD plus 10% serum at 35 °C for 3 hrs, observed by microscopy.

**Figure S4. Cell morphology of Eaf1$^{K173R}$, Eaf1$^{K173Q}$ and *brg1* carring Eaf1$^{K173R}$ mutant induced in different media.** An overnight culture of wild-type, Eaf1$^{K173R}$, Eaf1$^{K173Q}$, *brg1* and *brg1* carring Eaf1$^{K173R}$ was induced at 37 °C in YPD for 3 hrs, YPD with 10% serum for 3 hrs, YPD with 5 nM rapamycin for 4 hrs, YEP with 2% GlcNAc for 4 hrs or Lee's with 2% mannitol for 6 hrs. Cell-morpha were observed and photographed.

**Figure S5. Sequence alignment around the acetylable lysine in Eaf1**

**orthologues across eukaryotes.** The acetylable residue K173 is conserved in Eaf1 orthologues among polymorphic fungi, but is occupied by a non-acetylable residue arginine (R) in non-dimorphic yeast-like fungi or glutamine (Q) in filamentous fungi. The lysine residue is neither conserved among Eaf1 orthologues from protista, nor the Eaf1 orthologues from higher eukaryotes or p400-like proteins.

**Figure S1**

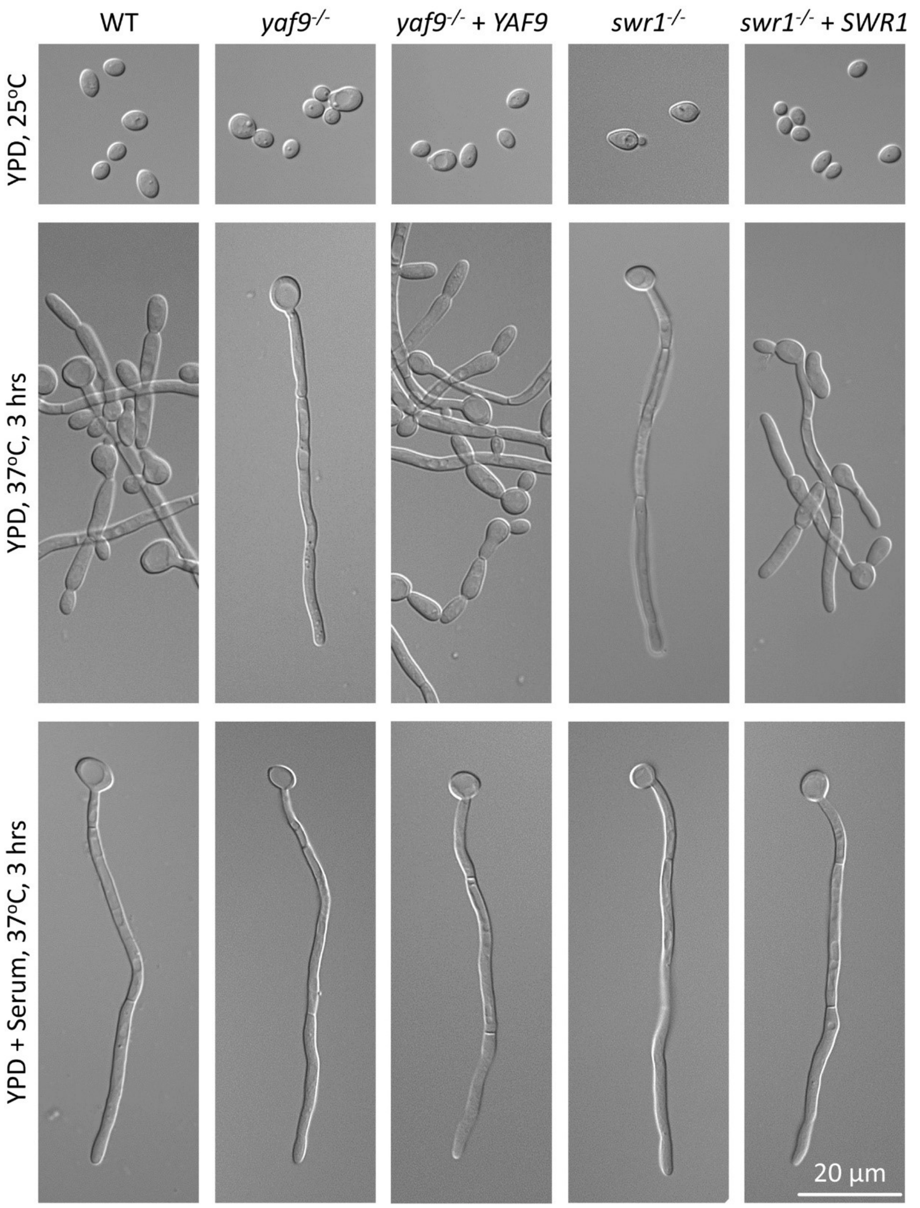
WT
yaf9-/-
yaf9-/- + YAF9
swr1-/-
swr1-/- + SWR1
YPD, 25oC
YPD, 37oC, 3 hrs
YPD + Serum, 37oC, 3 hrs
20 µm

**Figure S2**

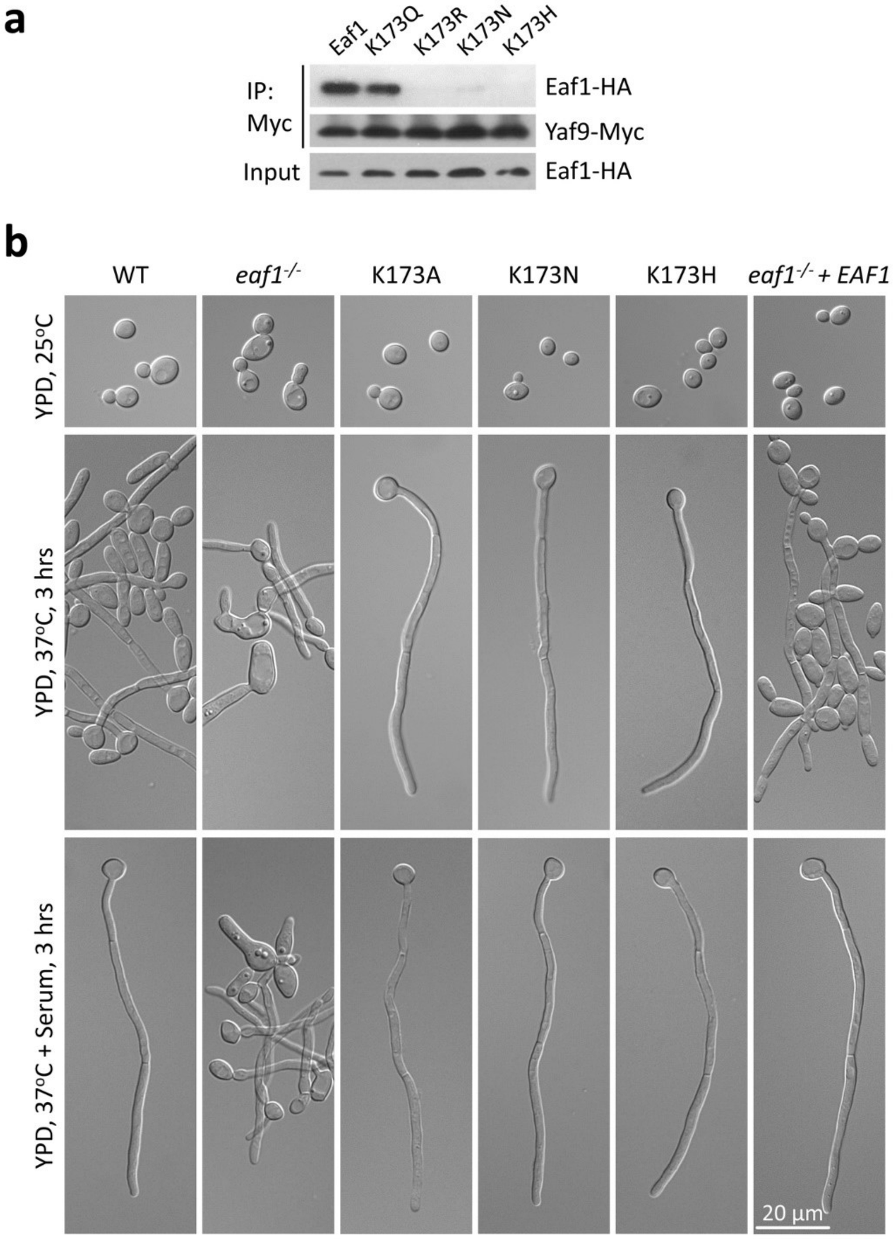
a
Eaf1
K173Q
K173R
K173N
K173H
IP:
Myc
Input
Eaf1-HA
Yaf9-Myc
Eaf1-HA
b
WT
eaf1-/-
K173A
K173N
K173H
eaf1-/- + EAF1
YPD, 25°C
YPD, 37°C, 3 hrs
YPD, 37°C + Serum, 3 hrs
20 μm

**Figure S3**

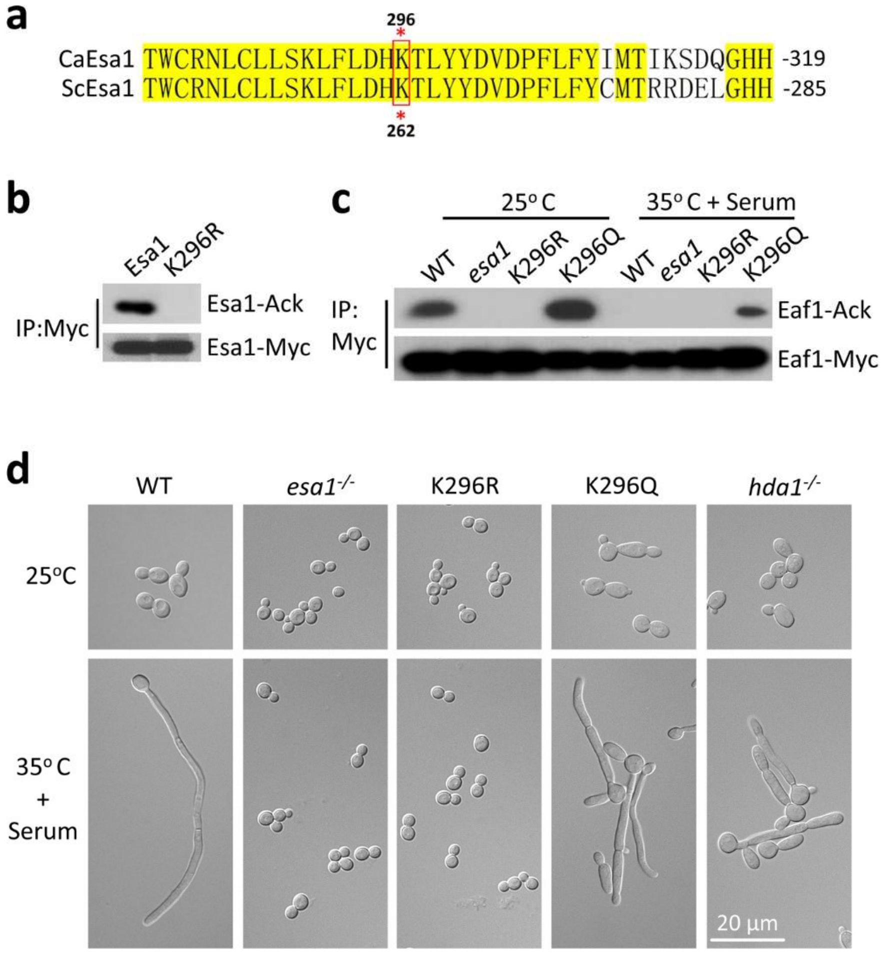
a
296
CaEsa1 TWCRNLCLLSKLFLDHKTLYYDVDPFLFYIMTIKSDQGHH -319
ScEsa1 TWCRNLCLLSKLFLDHKTLYYDVDPFLFYCMTRRDELGHH -285
262
b
Esa1
K296R
IP:Myc
Esa1-Ack
Esa1-Myc
c
25° C
35° C + Serum
WT
esa1
K296R
K296Q
WT
esa1
K296R
K296Q
IP:
Myc
Eaf1-Ack
Eaf1-Myc
d
WT
esa1-/-
K296R
K296Q
hda1-/-
25°C
35° C
+
Serum
20 μm

**Figure S4**

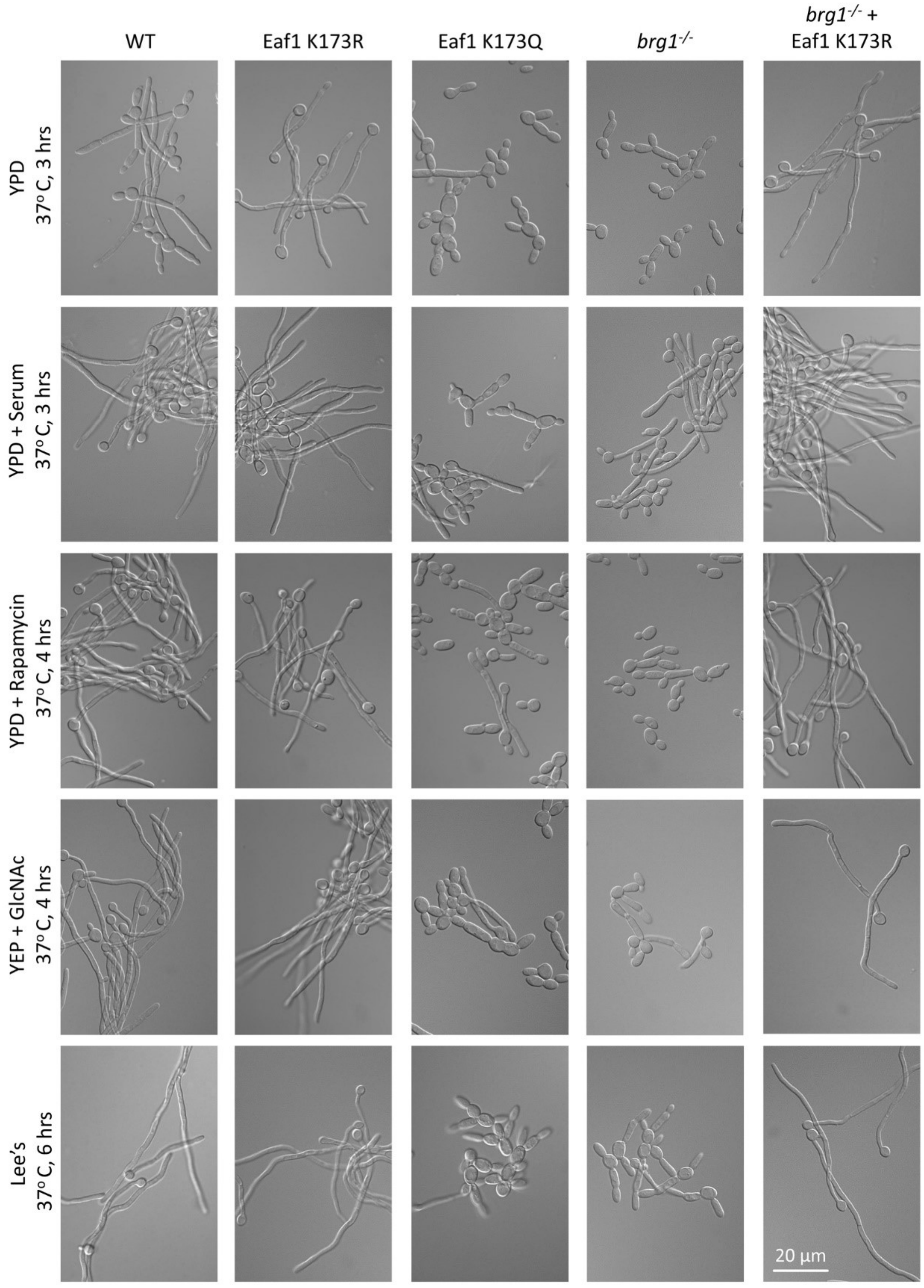

WT
Eaf1 K173R
Eaf1 K173Q
*brg1*$^{-/-}$
*brg1*$^{-/-}$ +
Eaf1 K173R
YPD
37º C, 3 hrs
YPD + Serum
37º C, 3 hrs
YPD + Rapamycin
37º C, 4 hrs
YEP + GlcNAc
37º C, 4 hrs
Lee's
37º C, 6 hrs
20 µm

## Figure S5

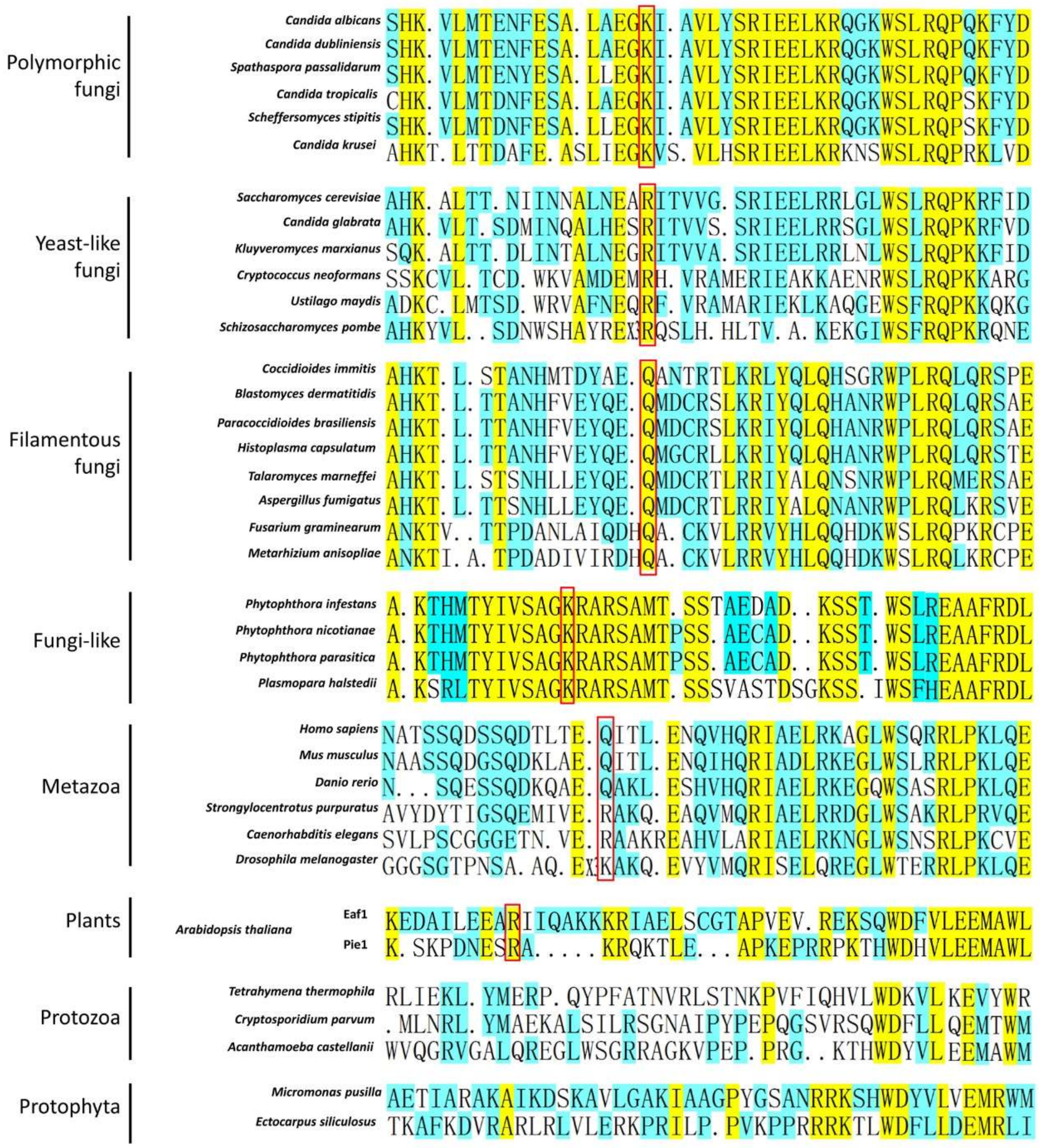
Polymorphic fungi
Candida albicans SHK. VLMTENFESA. LAEGKI. AVLYSRIEELKRQGKWSLRQPQKFYD
Candida dubliniensis SHK. VLMTENFESA. LAEGKI. AVLYSRIEELKRQGKWSLRQPQKFYD
Spathaspora passalidarum SHK. VLMTENYESA. LLEGKI. AVLYSRIEELKRQGKWSLRQPQKFYD
Candida tropicalis CHK. VLMTDNFESA. LAEGKI. AVLYSRIEELKRQGKWSLRQPSKFYD
Scheffersomyces stipitis SHK. VLMTDNFESA. LLEGKI. AVLYSRIEELKRQGKWSLRQPSKFYD
Candida krusei AHKT. LTTDAFE. ASLIEGKVS. VLHSRIEELKRKNSWSLRQPRKLVD
Yeast-like fungi
Saccharomyces cerevisiae AHK. ALTT. NIINNALNEARITVVG. SRIEELRRLGLWSLRQPKRFID
Candida glabrata AHK. VLT. SDMINQALHESRITVVS. SRIEELRRSGLWSLRQPKRFVD
Kluyveromyces marxianus SQK. ALTT. DLINTALNEGRITVVA. SRIEELRRLNLWSLRQPKKFID
Cryptococcus neoformans SSKCVL. TCD. WKVAMDEMRH. VRAMERIEAKKAENRWSLRQPKKARG
Ustilago maydis ADKC. LMTSD. WRVAFNEQRF. VRAMARIEKLKAQGEWSFRQPKKQKG
Schizosaccharomyces pombe AHKYVL. . SDNWSHAYREXRQSLH. HLTV. A. KEKGIWSFRQPKRQNE
Filamentous fungi
Coccidioides immitis AHKT. L. STANHMTDYAE. QANTRTLKRLYQLQHSGRWPLRQLQRSPE
Blastomyces dermatitidis AHKT. L. TTANHFVEYQE. QMDCRSLKRIYQLQHANRWPLRQLQRSAE
Paracoccidioides brasiliensis AHKT. L. TTANHFVEYQE. QMDCRSLKRIYQLQHANRWPLRQLQRSAE
Histoplasma capsulatum AHKT. L. TTANHFVEYQE. QMGCRLLKRIYQLQHANRWPLRQLQRSTE
Talaromyces marneffei AHKT. L. STSNHLLEYQE. QMDCRTLRRIYALQNSNRWPLRQMERSAE
Aspergillus fumigatus AHKT. L. TTSNHLLEYQE. QMDCRTLRRIYALQNANRWPLRQLKRSVE
Fusarium graminearum ANKTV. . TTPDANLAIQDHQA. CKVLRRVYHLQQHDKWSLRQPKRCPE
Metarhizium anisopliae ANKTI. A. TPDADIVIRDHQA. CKVLRRVYHLQQHDKWSLRQLKRCPE
Fungi-like
Phytophthora infestans A. KTHMTYIVSAGKRARSAMT. SSTAEDAD. . KSST. WSLREAAFRDL
Phytophthora nicotianae A. KTHMTYIVSAGKRARSAMTPSS. AECAD. . KSST. WSLREAAFRDL
Phytophthora parasitica A. KTHMTYIVSAGKRARSAMTPSS. AECAD. . KSST. WSLREAAFRDL
Plasmopara halstedii A. KSRLTYIVSAGKRARSAMT. SSSVASTDSGKSS. IWSFHEAAFRDL
Metazoa
Homo sapiens NATSSQDSSQDTLTE. QITL. ENQVHQRIAELRKAGLWSQRRLPKLQE
Mus musculus NAASSQDGSQDKLAE. QITL. ENQIHQRIADLRKEGLWSLRRLPKLQE
Danio rerio N. . . SQESSQDKQAE. QAKL. ESHVHQRIAELRKEGQWSASRLPKLQE
Strongylocentrotus purpuratus AVYDYTIGSQEMIVE. RAKQ. EAQVMQRIAELRRDGLWSAKRLPRVQE
Caenorhabditis elegans SVLPSCGGGETN. VE. RAAKREAHVLARIAELRKNGLWSNSRLPKCVE
Drosophila melanogaster GGGSGTPNSA. AQ. EXKAKQ. EVYVMQRISELQREGLWTERRLPKLQE
Plants
Arabidopsis thaliana
Eaf1 KEDAILEEARIIQAKKKRIAELSCGTAPVEV. REKSQWDFVLEEMAWL
Pie1 K. SKPDNESRA. . . . . KRQKTLE. . . APKEPRRPKTHWDHVLEEMAWL
Protozoa
Tetrahymena thermophila RLIEKL. YMERP. QYPFATNVRLSTNKPVFIQHVLWDKVL KEVYWR
Cryptosporidium parvum . MLNRL. YMAEKALSILRSGNAIPYPEPQGSVRSQWDFLL QEMTWM
Acanthamoeba castellanii WVQGRVGALQREGLWSGRRAGKVPEP. PRG. . KTHWDYVL EEMAWM
Protophyta
Micromonas pusilla AETIARAKAIKDSKAVLGAKIAAGPYGSANRRKSHWDYVLVEMRWM
Ectocarpus siliculosus TKAFKDVRARLRLVLERKPRILP. PVKPPRRRKTLWDFLLDEMRLI

## Table S1. *Candida albicans* strains used in this study

| Strain | Genotype | Source |
|---|---|---|
| SC5314 | Wild type | [1] |
| CAI4 | *ura3::λ imm434/ ura3:: λ imm434* | [1] |
| BWP17 | *ura3::λimm434/ura3::λ imm434 his1::hisG/his1::hisG arg4::hisG/arg4::hisG* | [2] |
| CWX1 | *ura3::λimm434/ura3::λimm434 his1::hisG/his1::hisG arg4::hisG/arg4::hisG ESA1/esa1::HIS1* | [3] |
| CWX2 | *ura3::λimm434/ura3::λ imm434 his1::hisG/his1::hisG arg4::hisG/arg4::hisG esa1::HIS1/esa1 ::hisG-URA3-hisG* | [3] |
| CWX3 | *ura3::λimm434/ura3::λ imm434 his1::hisG/his1::hisG arg4::hisG/arg4::hisG esa1::HIS1/esa1 ::hisG* | [3] |
| CLY3 | *ura3::λ imm434/ura3::λ imm434 yng2:: hisG /yng2::hisG-URA3-hisG* | [4] |
| CLY4 | *ura3::λ imm434/ura3::λ imm434 yng2:: hisG /yng2::hisG* | [4] |
| CWX10 | *ura3::λimm434/ura3::λimm434 his1::hisG/his1::hisG arg4::hisG/arg4::hisG SWR1/swr1::HIS1* | This study |
| CWX11 | *ura3::λimm434/ura3::λimm434 his1::hisG/his1::hisG arg4::hisG/arg4::hisG swr1::HIS1/swr1 ::hisG-URA3-hisG* | This study |
| CWX12 | *ura3::λimm434/ura3::λimm434 his1::hisG/his1::hisG arg4::hisG/arg4::hisG swr1::HIS1/swr1 ::hisG* | This study |
| CWX13 | *ura3::λimm434/ura3::λimm434 his1::hisG/his1::hisG arg4::hisG/arg4::hisG EAF1/eaf1::HIS1* | This study |
| CWX14 | *ura3::λimm434/ura3::λimm434 his1::hisG/his1::hisG arg4::hisG/arg4::hisG eaf1::HIS1/eaf1 ::hisG-URA3-hisG* | This study |
| CWX15 | *ura3::λimm434/ura3::λimm434 his1::hisG/his1::hisG arg4::hisG/arg4::hisG eaf1::HIS1/eaf1 ::hisG* | This study |
| CSC1 | *ura3::λimm434/ura3::λimm434 his1::hisG/his1::hisG arg4::hisG/arg4::hisG eaf7::HIS1/eaf7 ::ARG4* | [4] |
| CWX16 | *ura3::λimm434/ura3::λimm434 his1::hisG/his1::hisG arg4::hisG/arg4::hisG bdf1::HIS1/bdf1 ::hisG-URA3-hisG* | This study |
| CWX17 | *ura3::λimm434/ura3::λimm434 his1::hisG/his1::hisG arg4::hisG/arg4::hisG bdf1::HIS1/bdf1 ::hisG* | This study |
| CWX18 | *ura3::λimm434/ura3::λimm434 his1::hisG/his1::hisG arg4::hisG/arg4::hisG YAF9/yaf9::HIS1* | This study |
| CWX19 | *ura3::λimm434/ura3::λimm434 his1::hisG/his1::hisG arg4::hisG/arg4::hisG yaf9::HIS1/yaf9 ::hisG-URA3-hisG* | This study |
| CWX20 | *ura3::λimm434/ura3::λimm434 his1::hisG/his1::hisG arg4::hisG/arg4::hisG yaf9::HIS1/yaf9 ::hisG* | This study |
| CWX21 | *ura3::λimm434/ura3::λimm434 his1::hisG/his1::hisG arg4::hisG/arg4::hisG htz1::HIS1/htz1 ::hisG-URA3-hisG* | This study |
| CWX22 | *ura3::λimm434/ura3::λimm434 his1::hisG/his1::hisG arg4::hisG/arg4::hisG htz1::HIS1/htz1 ::hisG* | This study |
| CWX23 | *ura3::λimm434/ura3::λimm434 his1::hisG/his1::hisG arg4::hisG/arg4::hisG* | This study |

| | | |
|---|---|---|
| | *hda1::HIS1/hda1 ::hisG-URA3-hisG* | |
| CWX24 | *ura3::λimm434/ura3::λimm434 his1::hisG/his1::hisG arg4::hisG/arg4::hisG hda1::HIS1/hda1 ::hisG* | This study |
| CWX25 | *ura3::λimm434/ura3::λimm434 his1::hisG/his1::hisG arg4::hisG/arg4::hisG rpd3::HIS1/rpd3::hisG-URA3-hisG* | This study |
| CWX26 | *ura3::λimm434/ura3::λimm434 his1::hisG/his1::hisG arg4::hisG/arg4::hisG rpd3::HIS1/rpd3::hisG* | This study |
| CWX27 | *ura3::λimm434/ura3::λimm434 his1::hisG/his1::hisG arg4::hisG/arg4::hisG rpd31::HIS1/rpd31::hisG-URA3-hisG* | This study |
| CWX28 | *ura3::λimm434/ura3::λimm434 his1::hisG/his1::hisG arg4::hisG/arg4::hisG rpd31::HIS1/rpd31::hisG* | This study |
| CWX29 | *ura3::λimm434/ura3::λimm434 his1::hisG/his1::hisG arg4::hisG/arg4::hisG eaf1::HIS1/eaf1*$^{K173R}$ *::URA3* | This study |
| CWX30 | *ura3::λimm434/ura3::λimm434 his1::hisG/his1::hisG arg4::hisG/arg4::hisG eaf1::HIS1/ eaf1*$^{K173Q}$ *::URA3* | This study |
| CWX31 | *ura3::λimm434/ura3::λimm434 his1::hisG/his1::hisG arg4::hisG/arg4::hisG eaf1::HIS1/eaf1*$^{K489R}$ *::URA3* | This study |
| CWX32 | *ura3::λimm434/ura3::λimm434 his1::hisG/his1::hisG arg4::hisG/arg4::hisG eaf1::HIS1/EAF1-YAF9::URA3* | This study |
| CWX33 | *ura3::λimm434/ura3::λimm434 his1::hisG/his1::hisG arg4::hisG/arg4::hisG yaf9::HIS1/yaf9 dYEATS::URA3* | This study |
| CWX34 | *ura3::λimm434/ura3::λimm434 his1::hisG/his1::hisG arg4::hisG/arg4::hisG esa1::HIS1/esa1*$^{K296R}$ *::URA3* | This study |
| CWX35 | *ura3::λimm434/ura3::λimm434 his1::hisG/his1::hisG arg4::hisG/arg4::hisG esa1::HIS1/ esa1*$^{K296Q}$ *::URA3* | This study |
| *brg1* | *his1/his1 leu2/leu2 arg4/arg4 URA3/ura3::λimm434 IRO1/iro1::λimm434 brg1::CdHIS1/brg1::CmLEU2* | [5] |
| CWX36 | *his1/his1 leu2/leu2 arg4/arg4 ura3::λimm434/ura3::λimm434 IRO1/ iro1::λimm434 brg1::CdHIS1/brg1::CmLEU2 eaf1::hisG/eaf1*$^{K173R}$ *::URA3* | This study |

## Table S2. Plasmids used in this study

| Plasmid | Description | Source |
|---|---|---|
| pCUB6 | Replacement of *S. cerevisiae URA3* by *C. albicans URA3* in pNKY50 | [2] |
| pSWR1-KO | 0.50 kb and 0.55 kb KO fragments of *C. albicans SWR1* and *hisG-URA3-hisG* in pBluescript SK(+) | This study |
| pEAF1-KO | 0.53 kb and 0.6 kb KO fragments of *C. albicans EAF1* and *hisG-URA3-hisG* in pBluescript SK+ | This study |
| pYAF9-KO | 0.55 kb and 0.50 kb KO fragments of *C. albicans YAF9* and *hisG-URA3-hisG* in pBluescript SK(+) | This study |
| pBDF1-KO | 0.56 kb and 0.53 kb KO fragments of *C. albicans BDF1* and *hisG-URA3-hisG* in pBluescript SK(+) | This study |
| pHTZ1-KO | 0.50 kb and 0.50 kb KO fragments of *C. albicans HTZ1* and *hisG-URA3-hisG* in pBluescript SK(+) | This study |
| pPR671 | *C. albicans ACT1* promoter,13×myc-FLAG, *HIS1*, for gene expression under *ACT1* promoter control at *RP10* locus in genome | [4] |
| p671-ARG4 | *C. albicans ACT1* promoter,13×myc-FLAG, *ARG4* | [3] |
| pPR673 | *C. albicans ACT1* promoter, 13×myc-FLAG, *URA3* | [4] |
| p671-YAF9 | 1.42 kb containing 5’ region and ORF of *YAF9* in p671, for *YAF9-MYC* expression from its own promoter at *YAF9* locus | This study |
| p673-YAF9 | 1.42 kb containing 5’ region and ORF of *YAF9* in p673 | This study |
| p671-ARG4-YAF9 | 1.42 kb containing 5’ region and ORF of *YAF9* in p671-ARG4 | This study |
| p671-EAF1 | 3.4 kb containing 5’ region and ORF of *EAF1* in p671 | This study |
| p673-EAF1 | 3.4 kb containing 5’ region and ORF of *EAF1* in p673 | This study |
| p671-ARG4-EAF1 | 3.4 kb containing 5’ region and ORF of *EAF1* in p671-ARG4 | This study |
| p671-SWR1 | 5.4 kb containing 5’ region and ORF of *SWR1* in p671 | This study |
| p673-SWR1 | 5.4 kb containing 5’ region and ORF of *SWR1* in p673 | This study |
| p671- ARG4-SWR1 | 5.4 kb containing 5’ region and ORF of *SWR1* in p671-ARG4 | This study |
| p671-ESA1 | 2.18 kb containing 5’ region and ORF of *ESA1* in p671 | This study |
| p673-ESA1 | 2.18 kb containing 5’ region and ORF of *ESA1* in p673 | This study |
| p671-ARG4-ESA1 | 2.18 kb containing 5’ region and ORF of *ESA1* in p671-ARG4 | This study |
| P673-HDA1 | 2.5 kb containing 5’ region and ORF of *HDA1* in p673 | This study |
| pBES116 | *C. albicans URA3* vector for integration at *ADE2* locus in genome | [6] |
| pBES116-YAF9 | 0.765 kb full length *YAF9* in pBES116 for *YAF9* expression from its own promoter at *YAF9* locus | This study |
| pBES116-YAF9 dYEATS | 0.549 kb Yaf9 fragment lacking YEATS domain in pBES116 | This study |

| | | |
|---|---|---|
| pBES116-EAF1 | 2.06 kb full length *EAF1* in pBES116 for *EAF1* expression from its own promoter | This study |
| pEAF1 muts | Eaf1 $^{K173R}$, Eaf1 $^{K173Q}$, Eaf1 $^{K173A}$, Eaf1 $^{K173H}$, Eaf1 $^{K173N}$ and Eaf1 $^{K489R}$ mutation in pBES116-EAF1 | This study |
| pBES116-ESA1 | 2.18 kb *ESA1* in pBES116 for *ESA1* expression from its own promoter | [3] |
| pEAF1-YAF9 | *EAF1* and *YAF9* fusion fragment in pBES116 for Eaf1-Yaf9 fusion expression from *EAF1* promoter | This study |
| pBA1 | *C. albicans ADH1* promoter in BES116,<br>for gene expression under *ADH1* promoter control at *ADE2* locus | [7] |
| pBA1-YAF9 | 0.765 kb full length *YAF9* in pBA1 for *YAF9* expression from *ADH1* promoter | This study |
| pEaf1-myc | Eaf1-Myc fusion in pBA1 | This study |
| pEaf1-myc muts | Lysine to arginine substitution at K173, K447, K478, K481, K482, K489 and K552 in pEaf1-myc | This study |
| pEsa1-myc | Esa1-Myc fusion in pBA1 | This study |
| pEsa1-K296R-myc | Lysine to arginine substitution at K296 in p Esa1-myc | |
| pEsa1-HA | Esa1 with 3HA at C-terminus from its own promoter, *URA3* | This study |
| pSwr1-HA | Swr1 with 3HA at C-terminus from its own promoter, *URA3* | This study |
| pYaf9-HA | Yaf9 with 3HA at C-terminus from its own promoter, *URA3* | This study |
| pEaf1-HA | Eaf1 with 3HA at C-terminus from its own promoter, *URA3* | This study |
| pEaf1-HA muts | Eaf1 $^{K173R}$, Eaf1 $^{K173Q}$ , Eaf1 $^{K173A}$ and Eaf1 $^{K489R}$ mutation in pEaf1-HA | This study |
| pHda1-HA-URA | Hda1 with 3HA at C-terminus from its own promoter, *URA3* | This study |
| pHda1-HA-HIS | Hda1 with 3HA at C-terminus from its own promoter, *HIS1* | This study |
| pGST-Eaf1 | 2.06 kb full length *EAF1* in pGEX-4T1 for GST-Eaf1 expression in *E. coli* | This study |
| pGST-Eaf1 muts | Eaf1 $^{K173R}$ and Eaf1 $^{K173Q}$ mutation in pGST-Eaf1 | This study |
| pMBP-Yaf9 | 0.765 kb full length *YAF9* in pSJ8 for MBP-Yaf9 expression in *E. coli* | This study |
| pMBP-dYEATS | 0.549 kb Yaf9 fragment lacking YEATS domain in pSJ8 | This study |
| pMBP-YEATS | 0.218 kb Yaf9 fragment containing YEATS domain in pSJ8 | This study |

**Table S3. Primers used in this study**

| Primers | Sequence | Purpose and features |
|---|---|---|
| F1 | 5'- ATTAGTAACACATCCCTCTCATATACCGCAATGGAGACCAG AAAATTTGGAAGACGGCAAgttttcccagtcacgacgtt | KO *SWR1* by PCR method |
| R1 | 5'- AACTTACCAAAGTAATCTGTAGTAAACTCTCCCTCTTGAATA ACAACGTTATCCAACTGTtgtggaattgtgagcggata | |
| F2 | 5'- tgca agatct ctgcag CTGCAGGAAAAGTATCCGTA | pSWR1-KO-5' region |
| R2 | 5'- tgca agatct GCTGGCTGGTTCGTTTCCCA | |
| F3 | 5'- cgat ggatcc GACTAGAAATACAGTGGCCA | pSWR1-KO-3' region |
| R3 | 5'- cgat gcatgc ctgcag GTTCAAGTACACAACGTGCT | |
| F4 | 5'-GTGGCGAAATACTTGGCACAGTCTCTAAAACCACATACGCCA ACCCAATGCCAAACACCAgttttcccagtcacgacgtt | KO *EAF1* by PCR method |
| R4 | 5'-AAAGAAATACAACGCCACCGAAATGGACCCGCCACAACCGC AACTATGAAAAGAAGTTTAtgtggaattgtgagcggata | |
| F5 | 5'- tgca agatct ctgcag CCAACCCTGCAAACCCTCTAA | pEAF1-KO-5' region |
| R5 | 5'- tgca agatctGGTTTGGTTAAGAAGAAGAAG | |
| F6 | 5'- cgat ggatccGTCAGACAACGACCTTAAACA | pEAF1-KO-3' region |
| R6 | 5'- cgat gcatgc ctgcagCTAACGGATAGTCTTGGGGGC | |
| F7 | 5'- CCAATGGTATAGATAGAGATGTTTTAAAGTAATAGGTAATTA GGATATGCAAATATAAATgttttcccagtcacgacgtt | KO *YAF9* by PCR method |
| R7 | 5'- CCCATGAATGATGATAGCACTAGTTCTAGTTCTAGTTCTAGTT CTAGTTGTTATTTTAAAtgtggaattgtgagcggata | |
| F8 | 5'-tgca agatct ctgcag GATAGAGATGTTTTAAAGTAA | pYAF9-KO-5' region |
| R8 | 5'-tgca agatct GAATGAGTTGATGACATGGAT | |
| F9 | 5'-cgat ggatcc GAAAAATTAGCCCTCCTACAA | pYAF9-KO-3' region |
| R9 | 5'-cgat gcatgc ctgcag GCACTAGTTCTAGTTCTAGTT | |
| F10 | 5'-CCATCAATTTATAATCTTTAATTAATAATTTAGAAAAAATTCCCT TTCTCATCAAACTGGgttttcccagtcacgacgtt | KO *BDF1* by PCR method |
| R10 | 5'GTCTCTCGTTTTTGTTGGAATCTTCGGAGTTTTGAAACCTTAAGCT TTTTTTTTTAATTTtgtggaattgtgagcggata | |
| F11 | 5'- tgca agatct ctgcag GCTTGTTAAGAATGCTATCA | pBDF1-KO-5' region |
| R11 | 5'- tgca agatct GATGTGAACTTTGTTGGTTA | |
| F12 | 5'- cgat ggatcc GGTCAGCAAGGGTCCGACAA | pBDF1-KO-3' region |
| R12 | 5'- cgat gcatgc ctgcag GGTTAATTGTTGTTTATCTA | |
| F13 | 5'- CCAAAATTATTGCACTGGACACCACCTCTGTTCACTGCAAAC CAATTACGGTTTGGGTGGCGCGGTGGCGGCCGCATCTTT | Endogenous Esa1-HA |
| R13 | 5'- CGATAAAAGTATTAGAATATGGGGAACATCCTATTAAATATA TATCTATATATATATTTAGGGGGATCCACTAGTTCTAGA | |
| F14 | 5'-GATGGCATTGGTCACATTGATGAGTATATGTTACGGTTTATTT CGGATGGATATTATTTACGCGGTGGCGGCCGCATCTTT | Endogenous Swr1-HA |
| R14 | 5'-GCAAGAGATTTTTATAAAAGAAAGGGCCATTTGCTTAATAT GTAATACAAGGAGTAACTAGGGGGATCCACTAGTTCTAGA | |
| F15 | 5'- ATGTCATCAACTCATTCAAGAAGAATAAAATTTGTATCGATAT CAGTACCTATATTATATCGCGGTGGCGGCCGCATCTTT | Endogenous Yaf9-HA |

| | | |
|---|---|---|
| R15 | 5'-TTATTGTAGGAGGGCTAATTTTTCTTGTTCGAGTAATTTATATTG ATTTCGTTGATTTTC GGGGGATCCACTAGTTCTAGA | |
| F16 | 5'- ATGAACGATACTTGGAGGTCTCGATTAAAGGAGATTCTGCATA TCACTGCAGACCCATTCCGCGGTGGCGGCCGCATCTTT | Endogenous Eaf1-HA |
| R16 | 5'-CTACTTACGCGTTGTTTGGCGACCCTGACATGGGCGATGCAGTT TGGTTTTTTTGGTGTG GGGGGATCCACTAGTTCTAGA | |
| F17 | 5'- gct ggatcc GACGAAAATTTTTCCTTGATA | Endogenous Esa1-myc |
| R17 | 5'- ggag acgcgt cg GATAATGGCTTTTCTGGCGAA | |
| F18 | 5'- gct ggatcc GAGATGAGTATAATACAATCA | Endogenous Swr1-myc |
| R18 | 5'- ggag acgcgt cg TAAATAATATCCATCCGAAATA | |
| F19 | 5'- gct ggatcc TTGATGAAAAATTATCTACTC | Endogenous Yaf9-myc |
| R19 | 5'- ggag acgcgt cgTTGTAGGAGGGCTAATTTTTCT | |
| F20 | 5'- gct ggatcc GACAATACCAACGACGAAGA | Endogenous Eaf1-myc |
| R20 | 5'- ggag acgcgt cg CGCGTTGTTTGGCGACCCTGA | |
| F21 | 5'-gat gcggccgc GACAATACCAACGACGAAGACA | pBES116-EAF1 |
| R21 | 5'-gat ggtacc TTACGCGTTGTTTGGCGACCC | |
| F22 | 5'-gat gcggccgc TTGATGAAAAATTATCTACTC | pBES116-YAF9 |
| R22 | 5'-gat ggtaccTCATGTAGGAGGGCTAATTTTTCT | |
| F23 | 5'-GTGACATTTAAATTACATGAAACTTATGAAAATCCA | pBES116-YAF9 dYEATS |
| R23 | 5'-ATGTAATTTAAATGTCACTCTTGAATGAGTTGATGACAT | |
| F24 | 5'-gat gcggccgcGACAATACCAACGACGAAGACA | pEAF1-YAF9 |
| R24 | 5'- TCAGGGTCGCCAAACAACGCG ATGTCATCAACTCATTCAAGA | |
| F25 | 5'-TCTTGAATGAGTTGATGACATCGCGTTGTTTGGCGACCCTGA | |
| R25 | 5'-gatggtaccTCATGTAGGAGGGCTAATTTTTCT | |
| F26 | 5'-ACAGTCTGCCAGACTTCATCTGCTCTCCATTGACG | Hda1-HA |
| R26 | 5'-CTGACTGACTGACTGCTGGTGGAAGTTTCAAAGCA | |
| F27 | 5'-CTTGCCTTACCTCGGTTTGAAAAATAAAAGAACGAGCCTA | |
| R27 | 5'-TGTGTACGAGTTGCAATCTTCGGAAGAGGAGTAGTCTTCA | |
| F28 | 5'-GTATGGCAAGAGACCATCAAACG | |
| R28 | 5'-TCAGCACAGGGATATGGGAAGTA | |
| F29 | 5'- gct ggatcc TTATAGAGAAGAATGACTCA | Hda1-Myc |
| R29 | 5'- ggag acgcgt cgATCTTCGGAAGAGGAGTAGT | |
| F30 | 5'-gatc agatct ATGGCGGTAGCAGAAATCAAA | pBA1-YAF9 |
| R30 | 5'-gatc atcgat TTACCACCCAAACCGTAATTG | |
| F31 | 5'-CAGTCAGTCAGTCAGGATAACGCAGGAAAGAACAT | PUC18 backbone |
| R31 | 5'-AGTCTGGCAGACTGTCTTAAGGGATCCAAGCTTCCC GGGTTCTTAGACGTCAGGTGGCA | |
| F32 | 5'-TGCAACTCGTACACAAGCGCGCCAATTAGATCCGTG | URA3 amplification |
| R32 | 5'-CCGAGGTAAGGCAAGTTCTTTCCTGCGTTATCCTG | |
| F33 | 5'-gatgaattcATGTCATCAACTCATTCAAGA | pMBP-Yaf9 |
| R33 | 5'-gatctcgagTCATGTAGGAGGGCTAATTTTTCT | |
| F34 | 5'-gctggatccATGAACGATACTTGGAGGTCT | pGST-Eaf1 |
| R34 | 5'-ggagacgcgtctaGTTGTTTGGCGACCCTGACAT | |
| F35 | 5'-CCAGTTACTTCTGGATCATC | *HWP1*, qRT PCR |

| | | |
|---|---|---|
| R35 | 5'-TCGGTACAAACACTGTTAGA | |
| F36 | 5'-TGGTGTTGCGTGGTCATTG | *EAF1*, qRT PCR |
| R36 | 5'-ATCGGTGGTTCTCTGGAGTC | |
| F37 | 5'-GCTCAGTACCTCGCCAGTT | *EAF1-YAF9*, qRT PCR |
| R37 | 5'-GTTCTGGTGGTGTTGTTGGTT | |
| F38 | 5'-GCTTTTGGTGTTTGACGAGTTTCT | *ACT1*, qRT PCR |
| R38 | 5'-GTGAGCCGGGAAATCTGTATAGTC | |

## Reference


1. Fonzi, W. A., Irwin, M. Y. Isogenic strain construction and gene mapping in Candida albicans. *Genetics* 1993; **134**:717-728.

2. Wilson, R. B., Davis, D., Mitchell, A. P. Rapid hypothesis testing with Candida albicans through gene disruption with short homology regions. *J. Bacteriol.* 1999; **181**:1868-1874.

3. Wang, X., Chang, P., Ding, J., Chen, J. Distinct and redundant roles of the two MYST histone acetyltransferases Esa1 and Sas2 in cell growth and morphogenesis of Candida albicans. *Eukaryot. Cell* 2013; **12**:438-449.

4. Lu, Y. *et al.* Efg1-mediated Recruitment of NuA4 to Promoters Is Required for Hypha-specific Swi/Snf Binding and Activation in Candida albicans. *Mol. Biol. Cell* 2008; **19**:4260-4272.

5. Homann, O. R., Dea, J., Noble, S. M., Johnson, A. D. A phenotypic profile of the Candida albicans regulatory network. *PLoS Genet.* 2009; **5**:e1000783.

6. Feng, Q., Summers, E., Guo, B., Fink, G. Ras signaling is required for serum-induced hyphal differentiation in Candida albicans. *J. Bacteriol.* 1999; **181**:6339-6346.

7. Cao, F. *et al.* The Flo8 transcription factor is essential for hyphal development and virulence in Candida albicans. *Mol. Biol. Cell* 2006; **17**:295-307.
